# Loss of ARID3A perturbs intestinal epithelial proliferation–differentiation ratio and regeneration

**DOI:** 10.1084/jem.20232279

**Published:** 2024-08-16

**Authors:** Nikolaos Angelis, Anna Baulies, Florian Hubl, Anna Kucharska, Gavin Kelly, Miriam Llorian, Stefan Boeing, Vivian S.W. Li

**Affiliations:** 1 Stem Cell and Cancer Biology Laboratory, The Francis Crick Institute, London, UK; 2 Bioinformatics and Biostatistics Science Technology Platform, The Francis Crick Institute, London, UK

## Abstract

Intestinal stem cells at the crypt divide and give rise to progenitor cells that proliferate and differentiate into various mature cell types in the transit-amplifying (TA) zone. Here, we showed that the transcription factor ARID3A regulates intestinal epithelial cell proliferation and differentiation at the TA progenitors. ARID3A forms an expression gradient from the villus tip to the upper crypt mediated by TGF-β and WNT. Intestinal-specific deletion of Arid3a reduces crypt proliferation, predominantly in TA cells. Bulk and single-cell transcriptomic analysis shows increased enterocyte and reduced secretory differentiation in the Arid3a cKO intestine, accompanied by enriched upper-villus gene signatures of both cell lineages. We find that the enhanced epithelial differentiation in the Arid3a-deficient intestine is caused by increased binding and transcription of HNF1 and HNF4. Finally, we show that loss of *Arid3a* impairs irradiation-induced regeneration with sustained cell death and reprogramming. Our findings imply that Arid3a functions to fine-tune the proliferation–differentiation dynamics at the TA progenitors, which are essential for injury-induced regeneration.

## Teaser

The transit-amplifying (TA) cells at the intestinal crypt have the capability to proliferate, differentiate, and undergo regeneration upon injury. Angelis et al. show that ARID3A functions to fine tune the proliferation–differentiation dynamics at the TA cells and regulate injury-induced regeneration.

## Introduction

The intestinal epithelium is one of the fastest renewing and regenerating tissues, and its high turnover in cell composition is facilitated by Lgr5^+^ intestinal stem cells (ISCs) residing at the intestinal crypts. ISCs divide to generate a daughter cell that will either self-renew to generate another stem cell or enter the transit-amplifying (TA) zone for subsequent lineage specification (Barker, 2014; Barker et al., 2007; Tetteh et al., 2015). Intestinal lineage decision takes place at cell positions +4/+5, where progenitor cells are located (Clevers, 2013). These progenitors can be broadly divided into two main subtypes, absorptive and secretory, which are highly plastic and are able to re-acquire stemness for tissue regeneration upon injury (Tetteh et al., 2016; van Es et al., 2012; Baulies et al., 2020b). The stem cell-to-daughter cell transition in the intestinal epithelium is a highly dynamic and plastic process. Maintenance and regulation of the stem cell pool are controlled both by an epithelial cellular niche as well as by the mucosal stromal microenvironment (Meran et al., 2017).

A variety of signaling cascades has been well-described in regulating ISC maintenance, fate decision, and terminal maturation. WNT and NOTCH signaling are the key drivers to maintain stem cell identity at the bottom of the crypt. At the +4/+5 progenitors, NOTCH dictates the initial absorptive versus secretory fate decision: “NOTCH-ON” promotes enterocyte differentiation, whilst “NOTCH-OFF” de-represses the master regulator ATOH1 to drive secretory lineage specification. We have recently identified that the transcription corepressors MTG8 and MTG16 are also key regulators expressed at the progenitors to facilitate this binary fate decision process by repressing ATOH1 transcription (Baulies et al., 2020a). After the initial lineage commitment process at +4/+5 cells, these progenitors will continue to proliferate and differentiate at the TA cells in the upper crypts and will eventually exit the cell cycle for terminal differentiation in the villi. Emerging evidence reveals that enterocytes, goblet cells, and tuft cells exhibit a broad zonation of their gene expression program along the crypt–villus axis to facilitate different functions, highlighting the complexity of the cellular differentiation process (Manco et al., 2021; Moor et al., 2018; Beumer et al., 2022). Whilst the initial lineage specification at +4/+5 cells has been extensively studied, the molecular control of proliferation and differentiation states at the TA zone has been largely overlooked in the past. Characterizing the TA cell regulation will help understand the regulation of intestinal epithelial cell type composition under homeostasis and injury-induced regeneration.

Here, we report the transcription factor A+T rich interaction domain 3a (ARID3A) as a novel regulator of intestinal homeostasis in controlling the proliferation and differentiation dynamics of the TA progenitor cells. ARID3A forms an expression gradient from the villus tip to progenitor cells at the crypts driven by TGF-b and WNT signaling. Loss of *Arid3a* inhibits crypt proliferation, perturbs the absorptive versus secretory cell differentiation, and increases the expression of villus-tip gene signatures, leading to reduced regenerative capacity upon irradiation-induced injury. Our findings reveal the hitherto unrecognized role of ARID3A in coordinating crypt cell proliferation–differentiation ratio that is important for intestinal epithelial cell type composition under homeostasis and injury-induced regeneration in the intestine.

## Results

### *Arid3a* forms an expression gradient from villus tip to TA progenitor cells

**Figure 1.**
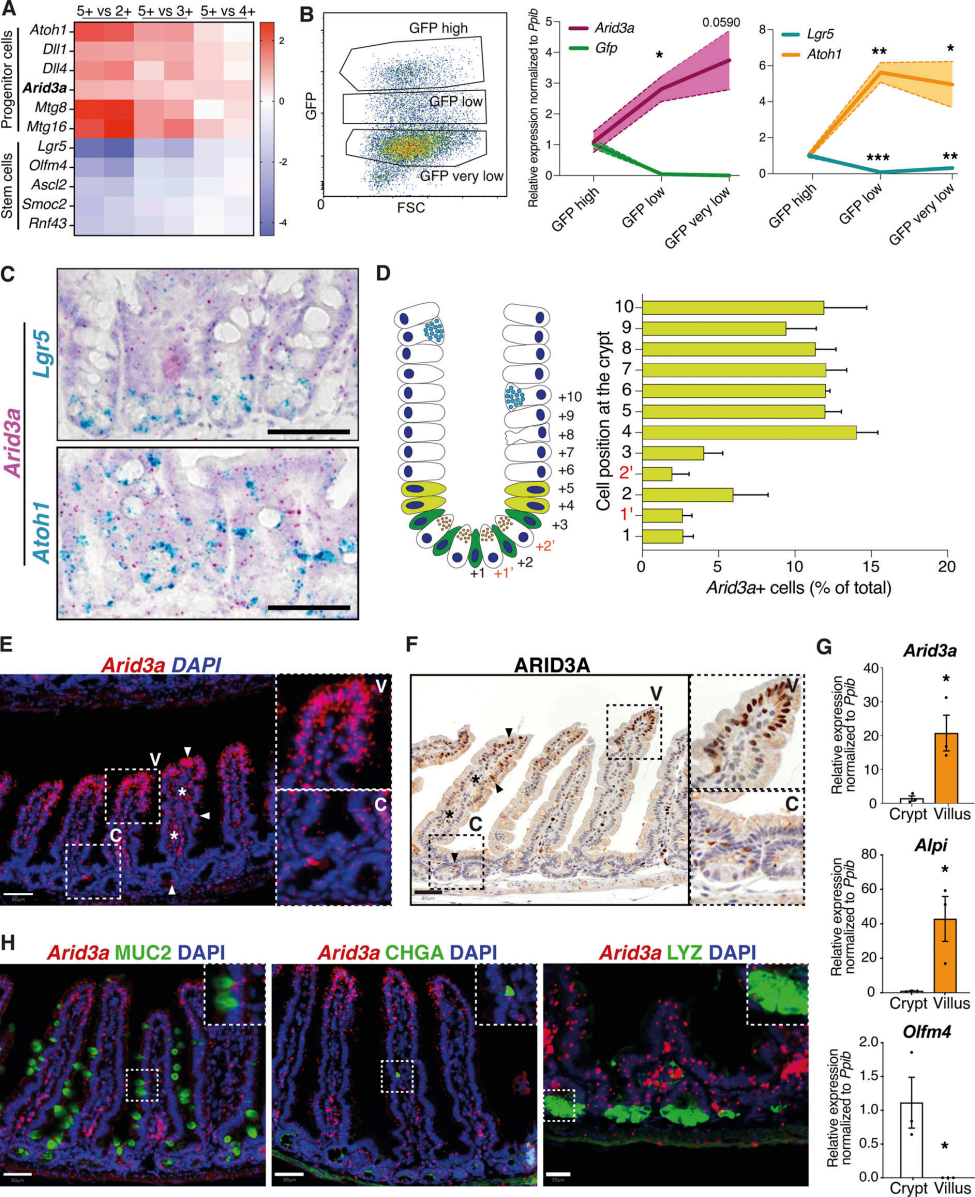
**ARID3A follows a gradient of expression across the crypt–villus axis. (A)** Re-analysis of differential gene expression between Lgr5-positive stem cells and their immediate progeny (GEO accession no. GSE36497) shows enrichment of *Arid3a* at the progenitor cells (Baulies et al., 2020a). **(B)** Left: Representative plot from FACS sorting of GFP^+^ cells from the Lgr5-EGFP-ires-CreERT2 mouse line (*N* = 3). Middle and right: qRT-PCR analysis of three FACS sorted populations representing ISCs (GFP high) and progenitor cells (GFP low and GFP very low). Three biologically independent animals (*N* = 3). Data represent mean ± SEM (light color area); *P < 0.05, **P < 0.01, ***P < 0.001, two-sided *t* test. **(C)** Dual RNAscope of *Arid3a* with *Lgr5* or Atoh1 at WT mouse small intestine. Each staining was performed on three different animals (*N* = 3). **(D)** Quantification of *Arid3a*^+^ cells per crypt position. Analysis was performed using the Arid3a/Lgr5 co-stain (*N* = 3). **(E)** RNAscope of *Arid3a* at WT mouse small intestine. Staining was performed on three different animals (*N* = 3). **(F)** Protein staining of ARID3A at WT mouse small intestine. Staining was performed on three different animals (*N* = 3). High-magnification images are shown in the insets. **(G)** qRT-PCR analysis of crypt and villus fractions. Three biologically independent animals (*N* = 3); *P < 0.05, **P < 0.01, ***P < 0.001, two-sided *t* test. **(H)** Combined *Arid3a* RNAscope scope staining with immunofluorescent protein staining of MUC2, CHGA, and LYZ. Each staining was performed on three different animals (*N* = 3). Scale bar, 50 μm for *Arid3a*/MUC2 and *Arid3a*/CHGA and 20 μm for *Arid3a*/LYZ.

We have previously identified a set of genes that are enriched at the +4/+5 progenitor cells compared to the *Lgr5*^+^ ISCs (Baulies et al., 2020a). Screening for transcription factors that are enriched at the +4/+5 cells identified *Arid3a* as a putative modulator of intestinal epithelial homeostasis (Fig. 1 A). Quantitative reverse transcription (qRT-PCR) analysis of Lgr5-GFP sorted cells isolated from a *Lgr5-EGFP-ires-CreERT2* intestinal crypts confirmed the enrichment at the GFP-low progenitors (Fig. 1 B). To further probe the localization of *Arid3a* at the crypt, we performed double RNAscope costaining of *Arid3a* with *Lgr5* (ISC marker) and *Atoh1* (secretory progenitor and Paneth cell marker). While *Arid3a* showed both overlapping and exclusive staining with *Lgr5* and *Atoh1*, the colocalization was minimal (Fig. 1 C). Quantification of the RNAscope data revealed that *Arid3a* not only was enriched at the +4/+5 cells as compared to the ISCs but its expression was maintained throughout the upper crypt (Fig. 1 D). Interestingly, expression analysis of ARID3A at both mRNA and protein levels showed a strong expression gradient from the villus tip to early progenitor cells at the crypt (Fig. 1, E and F). To confirm the enrichment of *Arid3a* at the villus compartment, we performed crypt–villus fractionation of mouse proximal small intestinal tissue followed by qRT-PCR analysis of the two compartments. As expected, the stem cell-specific marker *Olfm4* was enriched at the crypt fraction, while the enterocyte marker Alkaline phosphatase (*Alpi*) was enriched at the villus (Fig. 1 G). In accordance with our RNAscope data, *Arid3a* showed a 20-fold upregulation in the villus compared with the crypt (Fig. 1 G). Stromal expression of *Arid3a* was also detected (Fig. 1, E and F) since *Arid3a* is also expressed in B cells (Mowat and Agace, 2014; Ratliff et al., 2014).

Enterocytes are the most prominent type of intestinal epithelial cells exhibiting characteristic microvilli structures (McConnell et al., 2009). Careful examination of ARID3A protein staining confirmed the expression of ARID3A in brush border–bearing enterocytes (Fig. 1 F). We further tested if *Arid3a* is also expressed in secretory cells. By combing RNAscope staining of *Arid3a* and immunofluorescent staining to detect the protein levels of Mucin 2 (MUC2), Chromogranin A (CHGA), and Lysozyme (LYZ), we confirmed that *Arid3a* is also expressed in goblet, enteroendocrine, and Paneth cells, respectively (Fig. 1 H).

### WNT and TGF-β regulate the expression of *Arid3a* in epithelial cells

**Figure 2.**
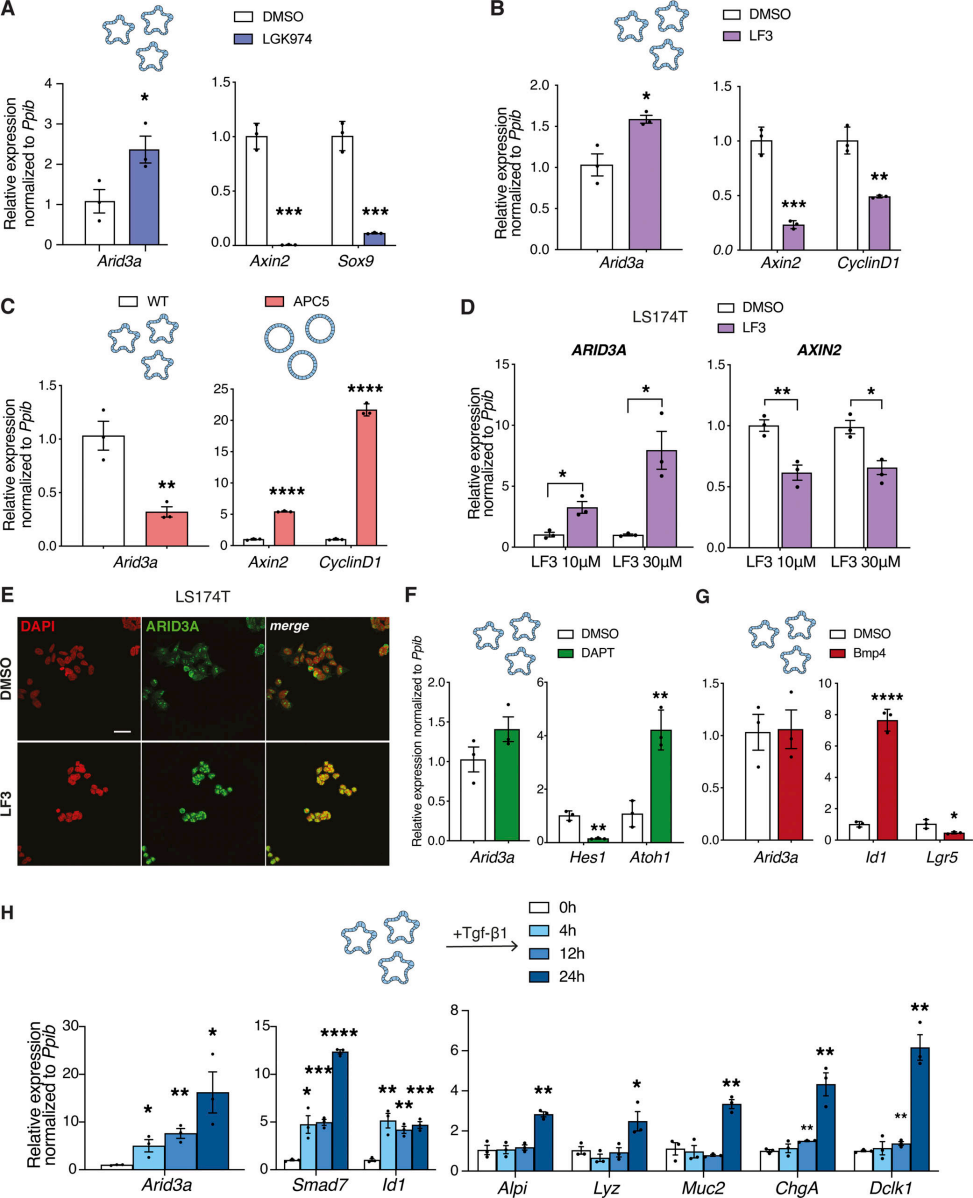
**WNT and TGF-β signaling fine-tune expression of Arid3a in the small intestinal epithelium. (A and B)** qRT-PCR analysis of WT organoids treated with LGK974 inhibitor (A) and LF3 inhibitor (B) for 48 h. Organoids were established from three biologically independent animals per group (*N* = 3). **(C)** qRT-PCR analysis of WT versus APC5 mutant organoids. Three independent experiments were performed (*n* = 3). **(D)** qRT-PCR analysis of LS174T cells treated with two different doses of LF3. Three independent experiments were performed (*n* = 3). **(E)** Immunofluorescence staining for ARID3A upon LF3 treatment of LS174T cells. Scale bar, 100 μm. Three independent experiments were performed (*n* = 3). **(F and G)** qRT-PCR analysis of WT organoids treated with DAPT inhibitor for 48 h (F) and recombinant BMP4 for 4 h (G). Organoids were established from three biologically independent animals per group (*N* = 3). **(H)** qRT-PCT analysis of WT organoids was treated with recombinant TGF-β1 for 4, 12, and 24 h. Analysis included *Arid3a*, *TGF-β* target genes, and markers of differentiation. Organoids were established from three biologically independent animals per group (*N* = 3). Data represent mean ± SEM; *P < 0.05, **P < 0.01, ***P < 0.001, two-sided *t* test.

Next, we sought to characterize the driver of *Arid3a* expression gradient. *Arid3a* forms an expression gradient from the upper crypt in a pattern opposite to the WNT gradient, implying that WNT signaling may regulate its expression. To test the regulatory role of WNT, ex vivo wild-type (WT) mouse intestinal organoids were treated with two different WNT inhibitors, LF3 and LGK974, for 48 h. Successful WNT inhibition was confirmed by downregulation of known WNT target genes, such as *Axin2*, *Sox9*, and *Cyclin D1* (Fig. 2, A and B). In contrast, *Arid3a* expression was upregulated upon treatment with either of the two inhibitors, suggesting a repressive role of WNT in *Arid3a* expression (Fig. 2, A and B). To validate the negative role of WNT in *Arid3a* expression, we further examined an independent WNT overactivation model using our previously published WNT-high mouse organoids carrying a truncated APC (APC5) (Novellasdemunt et al., 2017). Consistent with the observations earlier, *Arid3a* expression was downregulated, while the WNT targets *Axin2* and *Cyclin D1* were significantly increased in the WNT-high Apc5 organoids compared with WT control (Fig. 2 C).

Since WNT signaling regulates ISCs self-renewal and differentiation, the expression changes of *Arid3a* upon modulation of WNT in organoids might be caused indirectly by cell fate changes. To validate if WNT directly regulates *Arid3a* without change of cell fate, we switched to the human colorectal cancer cell line LS174T—with activated WNT signaling driven by β-catenin mutation—by treating them with two different doses of LF3 inhibitor (10 and 30 μΜ) for 24 h qRT-PCR analysis showed a robust upregulated expression of *ARID3A* and downregulation of WNT target *AXIN2* in both doses (Fig. 2 D). Immunofluorescent staining further confirmed upregulated expression of ARID3A at the protein level in LF3-treated LS174T cells (Fig. 2 E).

Since *Arid3a* is enriched at the early progenitor cells, we asked if its expression might be regulated by NOTCH signaling, the master regulator of early fate decision at the progenitors (Baulies et al., 2020b). Intestinal organoids were treated with the γ-secretase inhibitor DAPT (10 μM) for 48 h followed by qRT-PCR analysis. While DAPT treatment inhibited NOTCH target *Hes1* and de-repressed *Atoh1* expression, we did not observe any significant changes in *Arid3a* expression, indicating that its expression is independent of Notch signaling (Fig. 2 F).

Apart from WNT and NOTCH, TGF-β, and BMP signaling are also involved in intestinal homeostasis, while their dysregulation has been linked to cancer and other gastrointestinal diseases (Gudiño et al., 2021; Ihara et al., 2017; Tauriello et al., 2018; Zhang and Que, 2020). Interestingly, both TGF-β and BMP signaling form an expression gradient similar to that of *Arid3a*. To test whether the expression of *Arid3a* is regulated by the TGF-β superfamily, we treated mouse intestinal organoids with recombinant TGF-β1 and BMP4 ligands. As expected, BMP treatment activated its target gene *Id1* and inhibited ISC marker *Lgr5* expression (Auclair et al., 2007; Qi et al., 2017). However, the expression of *Arid3a* was unaffected by BMP (Fig. 2 G). In contrast, treatment of organoids with recombinant TGF-β1 for 4, 12, and 24 h revealed that *Arid3a* expression was increased over time, similar to the expression of TGF-β target genes *Smad7* and *Id1* (Fig. 2 H). It is important to note that the intestinal differentiation markers were not upregulated until 24 h after TGF-β induction, indicating that *Arid3a* expression was driven by TGF-β signaling directly rather than because of increased differentiation (Fig. 2 H).

### *Arid3a* regulates cell proliferation in intestinal TA progenitors

**Figure 3.**
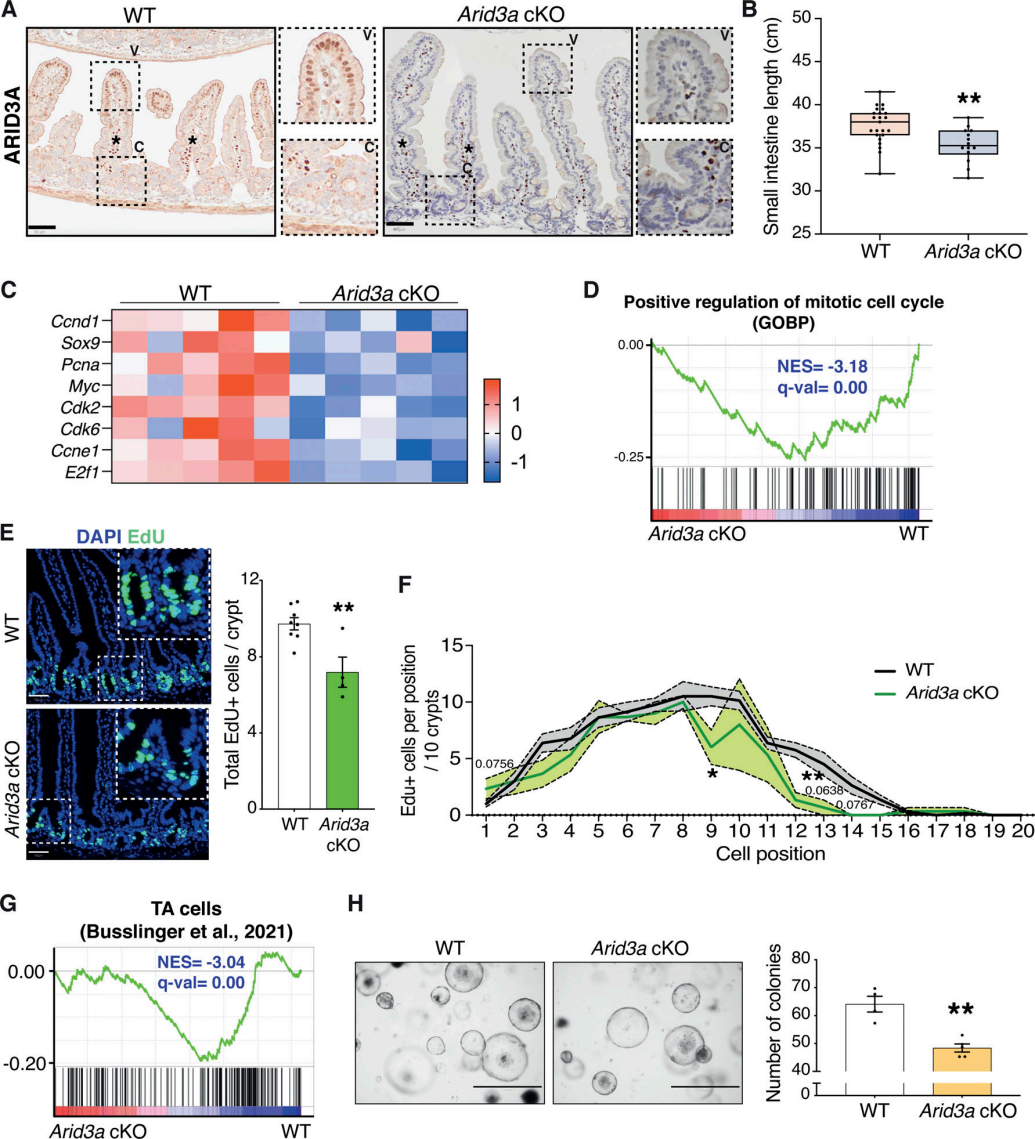
**Loss of *Arid3a* leads to loss of proliferative TA cells. (A)** ARID3A immunostaining of WT and *Arid3a* cKO mice. Representative images *N* = 3 of each group. Scale bar, 50 μm. High-magnification images are shown in the insets. **(B)** Differences in small intestinal length (cm) at 1 month after tamoxifen administration. *N* = 23 WT animals and *N* = 14 cKO animals. Box plot shows all datapoints from min to max. *P < 0.05, **P < 0.01, ***P < 0.001, two-sided *t* test. **(C)** Heatmap of RNA-seq data of representative proliferation markers. Z-scores are shown. **(D)** GSEA of GO biological process “positive regulation of mitotic cell cycle.” **(E)** EdU staining of WT and *Arid3a* cKO animals. Representative picture of *N* = 8 WT and *N* = 4 cKO animals. Scale bar, 50 μm. Quantification of total EdU-positive cells per crypt. Data represent mean ± SEM; *P < 0.05, **P < 0.01, ***P < 0.001, two-sided *t* test. **(F)** Quantification of EdU-positive cells based on their crypt position (per 10 crypts). Data represent mean ± SEM (light color area); *P < 0.05, **P < 0.01, ***P < 0.001, multiple two-sided *t* tests. **(G)** GSEA of previously published TA cells gene lists. **(H)** Images of WT and *Arid3a* cKO organoid formation assay and quantification of organoids per genotype at day 5 after isolation. Representative picture of *N* = 4 WT and *N* = 5 animals. Scale bar, 1,000 μm. Data represent mean ± SEM; *P < 0.05, **P < 0.01, ***P < 0.001, two-sided *t* test.

**Figure S1.**
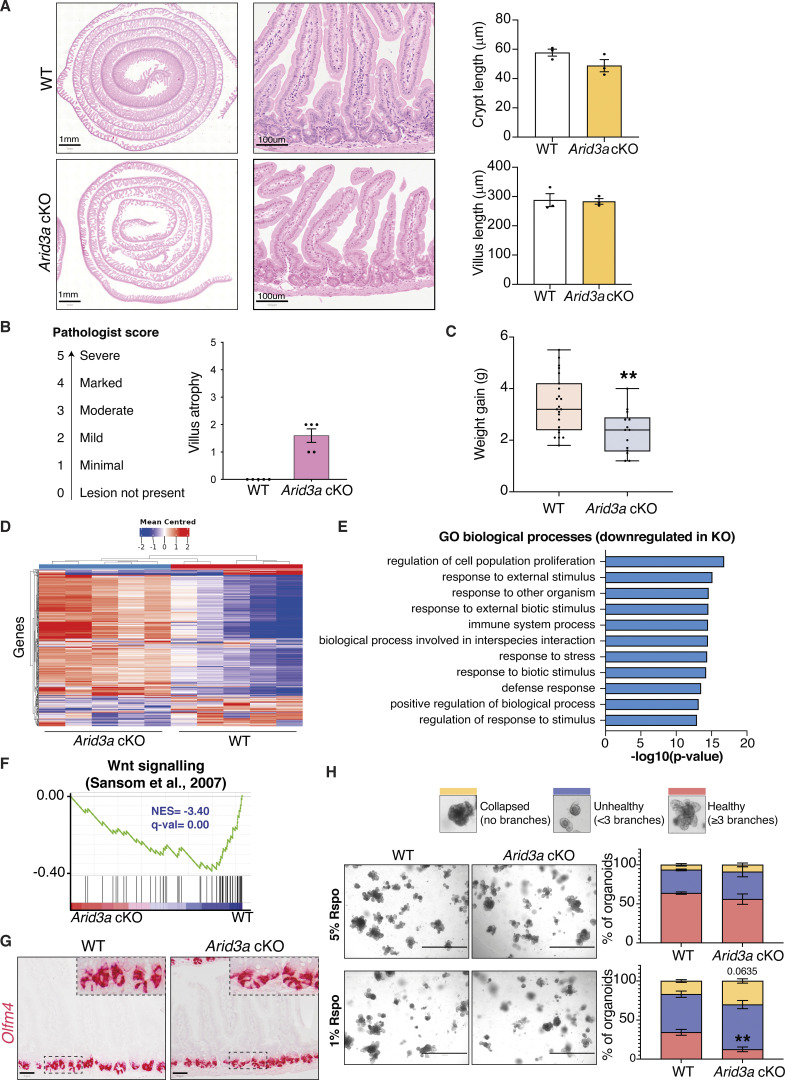
***Arid3a* cKO animals have reduced proliferative capacity. (A)** H&E staining of WT and *Arid3a* cKO mice at different magnifications. The length of crypts and villi were quantitated. 21 crypts or villi were measured per animal (*N* = 3). Representative images of *N* = 5 animals per experimental group. **(B)** Results of the pathologist’s report of WT and *Arid3a* cKO small intestinal tissue. *N* = 5 animals per experimental group. **(C)** Differences in weight gain (g) at 1 month after tamoxifen administration. *N* = 23 WT animals and *N* = 14 cKO animals. **(D)** Heatmap of blinded transcriptome-wide gene expression. The colors in the heatmap are a gene’s expression in a sample, relative to its average expression. **(E)** Top 10 downregulated GO biological processes based on RNA-seq analysis. FDR < 0.05 and fold change >1.5 cut-offs were applied. **(F)** GSEA of previously published WNT signaling gene list. **(G)** RNAscope staining of *Olfm4* in WT and *Arid3a* cKO mice. Representative picture of *N* = 3 per group. Scale bar, 50 μm. **(H)** Representative images and quantification of WT and *Arid3a* cKO organoids cultured in 5% or 1% Rspo CM. *N* = 8 WT and *N* = 4 Arid3a cKO organoid lines were used. Stacked data represent mean ± SEM *P < 0.05, **P < 0.01, ***P < 0.001, two-way ANOVA.

It has been previously reported that *Arid3a*-null mice exhibit embryonic lethality due to defects in hematopoiesis (Webb et al., 2011). In order to characterize the functional role of ARID3A in intestinal epithelial cells, we generated an in vivo conditional knockout mouse model, *Villin*Cre-ERT2^+/−^; *Arid3a*^fl/fl^ (*Arid3a* cKO), to delete *Arid3a* in *Villin*^+^ intestinal epithelial cells upon tamoxifen induction (el Marjou et al., 2004; Habir et al., 2017). Analysis of intestinal tissues 1 month post-tamoxifen administration confirmed complete abolishment of ARID3A expression at the epithelial cells, while the expression remained unchanged at stromal cells (Fig. 3 A). Hematoxylin and eosin (H&E) staining did not show any noticeable changes in the gross morphology of the *Arid3a*-depleted intestine (Fig. S1 A). However, semi-quantitative pathologist scoring revealed that *Arid3a* cKO animals exhibited minimal to mild villus atrophy (Fig. S1, A and B). Moreover, *Arid3a* cKO mice were found to have a shorter small intestine (mean = 35.35 cm) when compared with WT (mean = 37.60 cm) (Fig. 3 B). *Arid3a* cKO animals also gained significantly less weight (mean = 2.31 g) compared with WT animals (mean = 3.39 g) (Fig. S1 C).

To characterize the molecular changes caused by *Arid3a* deletion, unbiased RNA sequencing (RNA-seq) analysis was performed on the *Arid3a* cKO and WT intestine. Hierarchical clustering analysis showed that samples with the same genotypes readily clustered together (Fig. S1 D). Differential gene expression analysis revealed a total of 4,387 genes differentially expressed between the two groups (FDR cut-off < 0.05), with 2,413 genes upregulated and 1,974 downregulated in the *Arid3a* cKO intestine (Table S1). In particular, we observed a mild to moderate decrease of various cell cycle and cell proliferation markers in the *Arid3a*-depleted intestinal crypts (Fig. 3 C). Indeed, gene set enrichment analysis (GSEA) of mitotic cell cycle regulators indicated a strong downregulation in cKO intestine (Fig. 3 D), while Metacore analysis identified the regulation of cell population proliferation as the most significantly affected gene ontology (GO) biological process (Fig. S1 E). To assess the number of mitotically active cells in the crypts, animals were injected with a short pulse of 5-ehtynyl-2′deoxyuridine (EdU) to label cells undergoing de novo DNA synthesis or S-phase synthesis of the cell cycle. In accordance with our previous findings, *Arid3a* cKO intestine showed a reduction in the total number of proliferative cells per crypt compared to WT animals (WT mean = 9.73 cells; cKO mean = 7.2 cells) (Fig. 3 E). Interestingly, quantitation of EdU-positive cells revealed that reduced proliferation was mostly observed at cell positions 9–15 (counting from the crypt bottom), whereas EdU-labeling was largely unchanged at cell positions 1–5 (Fig. 3 F). This result suggests that *Arid3a* depletion inhibits proliferation predominantly at TA cells in the upper crypt whilst ISCs at the crypt base are mostly unaffected. Indeed, GSEA revealed a significant transcriptional downregulation of and TA cell gene signature (Busslinger et al., 2021) as well as in WNT signaling (Sansom et al., 2007) at *Arid3a* cKO crypts (Fig. 3 G and Fig. S1 F). Of note, RNAscope analysis showed no major difference in the expression of the ISC-specific marker *Olfm4* between WT and *Arid3a* cKO tissues, indicating that the number of stem cells was not affected upon *Arid3a* deletion (Fig. S1 G).

To validate the in vivo data, we generated ex vivo organoid cultures for functional analysis. Organoid formation analysis showed a significant decrease in organoid formation capacity in *Arid3a* cKO-derived crypts (WT mean = 64.1 organoids, cKO mean = 48.4 organoids) (Fig. 3 H). We further challenged the organoids by depleting one of the essential growth factors and WNT agonist, RSPONDIN (RSPO), in the culture medium to test the organoid dependency on exogenous WNT signal. Murine organoid cultures rely on supplementation of exogenous growth factors RSPO, EGF, and NOGGIN to survive (Sato et al., 2009). Under normal conditions, organoids were cultured in a medium containing 5% RSPO conditioned media (CM). Organoids derived from WT and cKO animals were assessed and quantified based on their morphologies: organoids with >3 buds were considered healthy, organoids with 1–3 buds were considered unhealthy and organoids that failed to bud or form cysts were considered collapsed. WT and cKO organoids did not exhibit any major morphological differences in the presence of 5% RSPO CM (Fig. S1 H). However, when organoids were challenged with a lower RSPO concentration (1%), a higher percentage of collapsed and a lower percentage of healthy organoids were observed (Fig. S1 H). The increased dependence on exogenous RSPO in the cKO organoids suggests a reduction of endogenous WNT signaling in the *Arid3a*-depleted crypt cells, which is consistent with the GSEA data observed in Fig. S3 E.

### Loss of *Arid3a* perturbs absorptive and secretory cell differentiation

**Figure 4.**
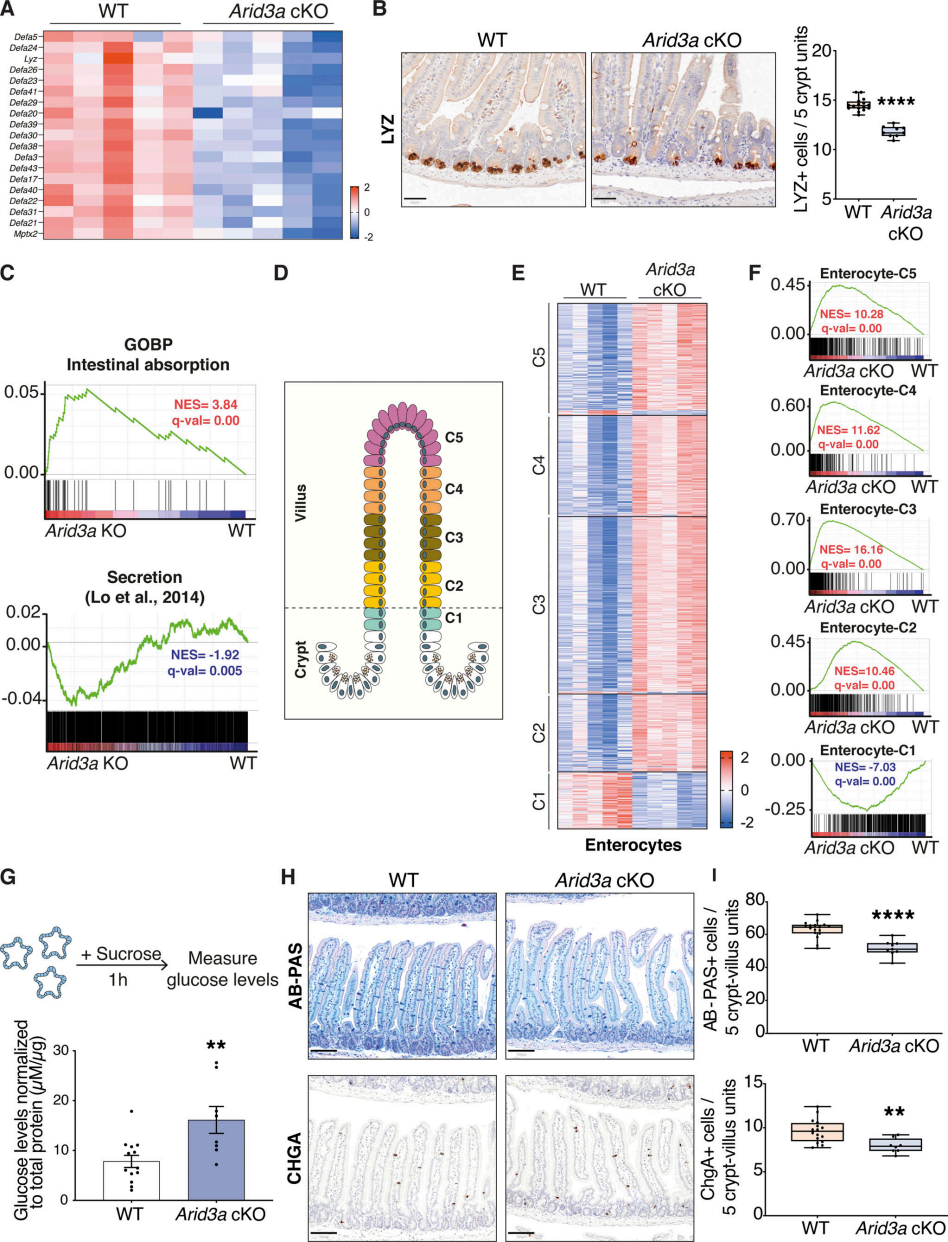
**Loss of Arid3a leads to upregulation of mid-villus and villus tip gene signatures of all lineages. (A)** Heatmap of RNA-seq data of representative Paneth cell markers. Z-scores are shown. **(B)** LYZ staining of WT and Arid3a cKO animals. Scale bar, 50 μm. Representative images and quantification of *N* = 16 WT and *N* = 9 KO animals. Box plot shows all datapoints from min to max. *P < 0.05, **P < 0.01, ***P < 0.001, two-sided *t* test. **(C)** GSEA of intestinal absorption and secretion-related genes. **(D)** Illustration of enterocyte zonation model as proposed by Moor et al. (2018). **(E)** Heatmap of differentially expressed genes of all enterocyte clusters based on RNA-seq data. Z-scores are shown. FDR cut-off <0.05. **(F)** GSEA of all genes included in the five enterocyte clusters (Moor et al., 2018). **(G)** Disaccharide assay: WT and *Arid3a* cKO organoids were treated with sucrose for 1 h. Graph shows absorbance levels of glucose normalized to total protein. Data represent mean ± SEM; *P < 0.05, **P < 0.01, ***P < 0.001, two-sided *t* test. **(H)** AB-PAS and CHGA staining of WT and Arid3a cKO animals. Scale bar, 100 μm. Representative images of *N* = 17 WT and *N* = 9 KO animals for AB-PAS and *N* = 16 WT and *N* = 8 KO animals for CHGA. **(I)** Quantification of AB-PAS and CHGA stainings. 25 crypt/villus units were counted for each animal. i.e., 425 WT and 225 Arid3a cKO crypt–villus units for AB-PAS staining; 400 WT and 200 Arid3a cKO crypt–villus units for ChgA staining. Box plot shows all datapoints from min to max. *P < 0.05, **P < 0.01, ***P < 0.001, two-sided *t* test.

Next, we asked if functional differentiation is affected in the cKO intestine. We first looked into the differentiation of Paneth cells, the only specialized epithelial cells that reside at the crypt base adjacent to ISCs and the main source of WNT ligands in ex vivo organoid culture (Baulies et al., 2020b). RNA-seq analysis revealed downregulation of various Paneth cell markers in *Arid3a* cKO intestine, including *Lyz1*, the newly discovered marker *Mptx2* (Haber et al., 2017), as well as a large number of antimicrobial peptides (α-defensins, *Defa*) secreted specifically from Paneth cells (Vandenbroucke et al., 2014) (Fig. 4 A). Immunostaining of LYZ further confirmed the reduction of Paneth cell numbers in the cKO intestine (WT mean = 14.53 cells/5 crypts, cKO mean = 11.82 cells/5 crypts) (Fig. 4 B). Paneth cells function as ISC niche by secretion of essential niche factors such as WNT ligands (Sato et al., 2011). Reduced Paneth cell numbers in Arid3a-deficient intestine may have shortened the WNT signal gradient in the crypt, leading to the observation of reduced WNT signature (Fig. S1 F) and organoid formation (Fig. 3 H and Fig. S1 H).

**Figure S2.**
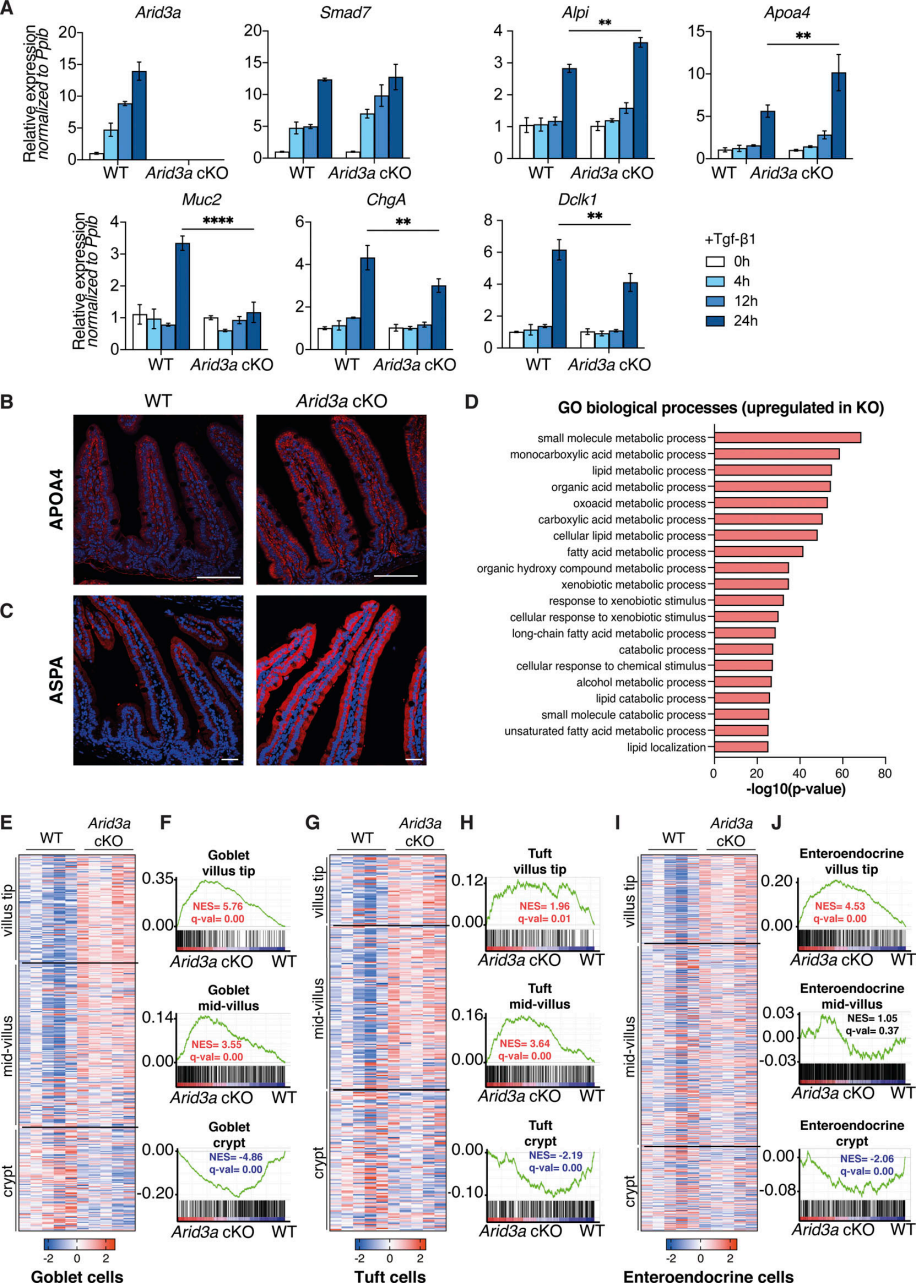
**Loss of *Arid3a* leads to disruption of enterocyte zonation. (A)** qRT-PCT analysis of WT and *Arid3a cKO* organoids treated with recombinant TGF-β1 for 4, 12, and 24 h. Organoids were established from three biologically independent animals per group (*N* = 3). Data represent mean ± SEM; **P < 0.01, ***P < 0.001, two-way ANOVA. **(B)** Immunofluorescence staining of APOA4. Representative images from *N* = 5 per experimental group. Scale bar, 100 μm. **(C)** Immunofluorescence staining of ASPA. Representative images from *N* = 3 per experimental group. Scale bar, 100 μm. **(D)** Top 20 upregulated GO biological processes based on RNA-seq analysis. FDR < 0.05 and fold change >1.5 cut-offs were applied. **(E)** Heatmap of RNA-seq data of goblet cell zonated and differentially expressed genes. Z-scores are shown. FDR cut-off <0.05. **(F)** GSEA of all zonated goblet cell genes. **(G)** Heatmap of RNA-seq data of tuft cell zonated and differentially expressed genes. Z-scores are shown. FDR cut-off <0.05. **(H)** GSEA of all zonated tuft cell genes. **(I)** Heatmap of RNA-seq data of enteroendocrine cell zonated and differentially expressed genes. Z-scores are shown. FDR cut-off <0.05. **(J)** GSEA of all zonated enteroendocrine cell genes. For E, G, and I, genes are shown based on their center of mass with crypt genes at the bottom of the heatmap and villus tip genes at the top (based on Manco et al. [2021]).

In adult intestinal crypts, ISCs give rise to progenitor cells at the TA zone that adopt either absorptive or secretory fate. Apart from Paneth cells, all other differentiated epithelial cells migrate up toward the villus after lineage commitment. GSEA showed significant enrichment of absorptive gene signature and an overall reduced secretion signature in the mutant intestine (Fig. 4 C), suggesting that Arid3a regulates the differentiation ratio between absorptive and secretory cells. To test if such altered differentiation is mediated via TGF-β signaling, we further treated WT and *Arid3a* cKO organoids with TGF-β. Quantitative RT-PCR analysis showed that loss of Arid3a inhibited TGF-β-induced secretory differentiation and enhanced TGF-β-induced enterocyte differentiation, supporting the notion that TGF-β-induced differentiation is dependent on ARID3A (Fig. S2 A).

Recent studies further revealed that the differentiated cells do not acquire their terminal identity at the TA zone. Rather, the committed cells were further zonated along the villi to carry out different functions (Manco et al., 2021; Moor et al., 2018). In particular, enterocyte zonation can be grouped into five distinct functional clusters across the crypt–villus axis (Moor et al., 2018) (Fig. 4 D). Interestingly, differential gene expression analysis and GSEA showed that Cluster 1 (early crypt enterocytes) was strongly enriched at WT animals, while the gene signatures of Clusters 2–5 were upregulated in *Arid3a* cKO intestines (Fig. 4, E and F). Immunostaining of the villus tip-enriched markers APOA4 and ASPA confirmed their upregulation and perturbed zonated expression in the cKO intestines (Fig. S2, B and C). It has been previously shown that genes in Cluster 2 are associated with mitochondrial activity, and Clusters 3–5 are associated with absorption, nutrient transport, brush border function, and cell adhesion (Moor et al., 2018). Interestingly, Metacore analysis of the significantly upregulated genes of the RNA-seq dataset (FDR < 0.05, fold change >1.5) showed that most of the top upregulated GO biological processes were related to an increase of various metabolic processes (Fig. S2 D), which are likely facilitated by the increased enterocyte absorptive gene signatures in Clusters 2–5. Furthermore, a disaccharidase functional assay confirmed an increased sucrose breakdown to glucose in the *Arid3a* cKO organoids, indicating higher enterocyte digestion activity in the *Arid3a*-depleted intestinal epithelium compared with WT control (Fig. 4 G).

Contrary to the enterocyte differentiation, secretory lineage appeared to be downregulated in the Arid3a-deficient intestine (Fig. 4 C). Apart from Paneth cells, goblet and enteroendocrine cells are the two most abundant secretory cell types in the intestinal epithelium throughout the villus. Indeed, staining of Alcian Blue/Periodic Acid-Schiff (AB-PAS) and chromogranin A (CHGA) showed minimal reduction of goblet and enteroendocrine cells, respectively, in the *Arid3a* cKO intestine (Fig. 4, H and I). Similar to enterocytes, several recent studies have reported the spatial differentiation dynamics of secretory cells (Beumer et al., 2018; Gehart et al., 2019; Manco et al., 2021). Goblet cells and tuft cells show a spatial expression program across the crypt–villus axis, similar to one the of enterocytes, while enteroendocrine cells show a more complex spatio-temporal migration pattern (Gehart et al., 2019; Manco et al., 2021). Interestingly, despite the observed overall reduced secretory cell differentiation, we noted a subtle but significant enrichment of the villus-tip gene expression program across all three secretory lineages upon *Arid3a* deletion (Fig. S2, E–J), suggesting that ARID3A may regulate terminal differentiation of secretory cells at the upper villus. Together, our data indicates that deletion of ARID3A impairs enterocytes versus secretory cell differentiation ratio and spatial gene signatures of the intestinal epithelium.

### Single-cell RNA sequencing reveals changes in enterocyte differentiation trajectory and cell cycle phase in stem-progenitor cells

**Figure S3.**
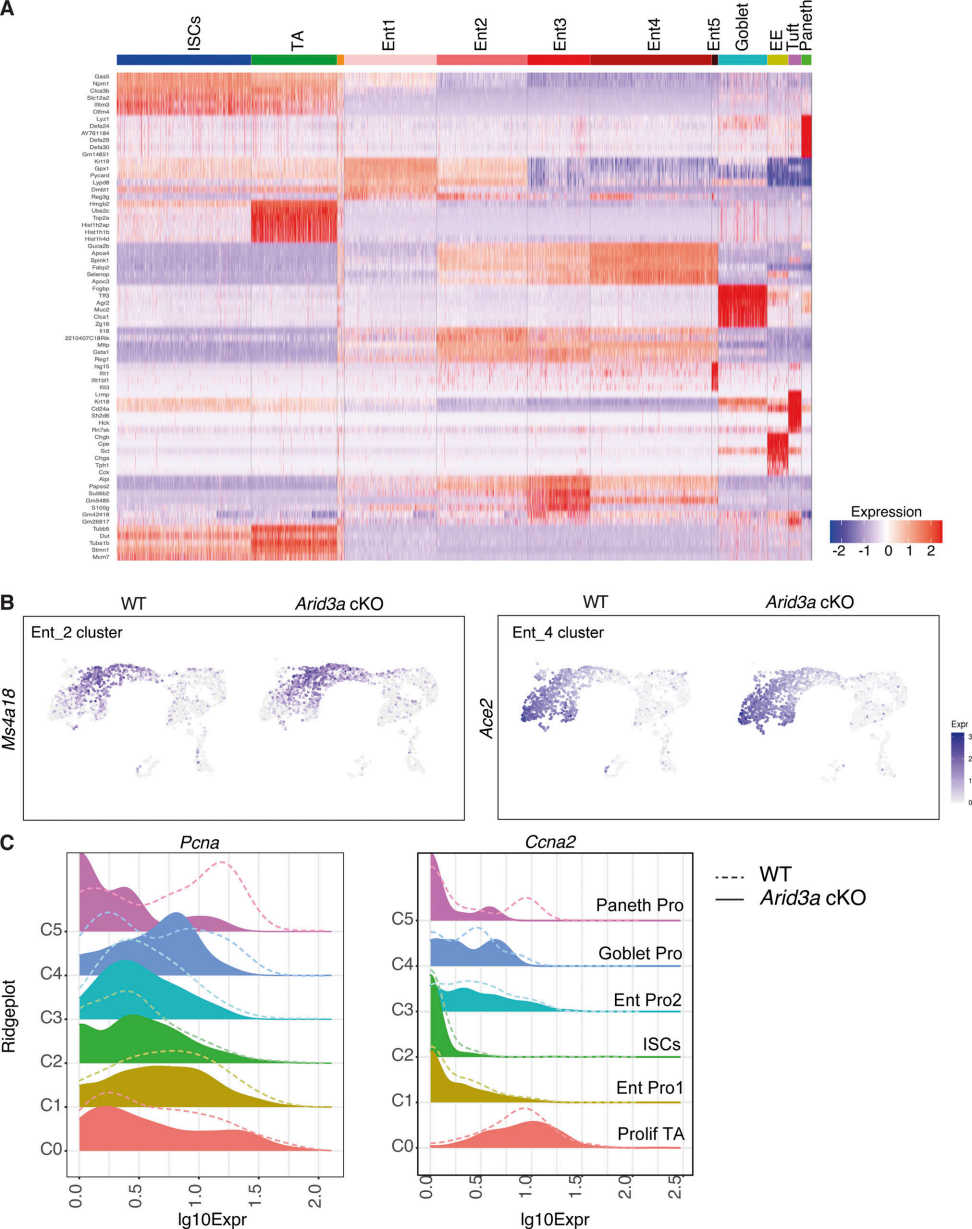
**Single-cell RNA-sequencing reveals changes in enterocyte differentiation trajectory and cell cycle phase in TA cells. (A)** Top: Cluster markers heatmap showing the most distinct marker genes in each cluster. Cluster annotation was based on identification of previously published genes within this list. Cluster annotated in orange included a very small number of cells with no specific enrichment for specific genes. This cluster was excluded from any further downstream analysis. **(B)** Feature plot of *Ms4a18* (enterocyte marker cluster 2) and *Ace2* (enterocyte marker cluster 5) expression on UMAP. **(C)** Ridgeplots showing log_10_ expression of *Pcna* and *Ccna2* genes in WT and *Arid3a* cKO for each crypt cell sub-population.

**Figure 5.**
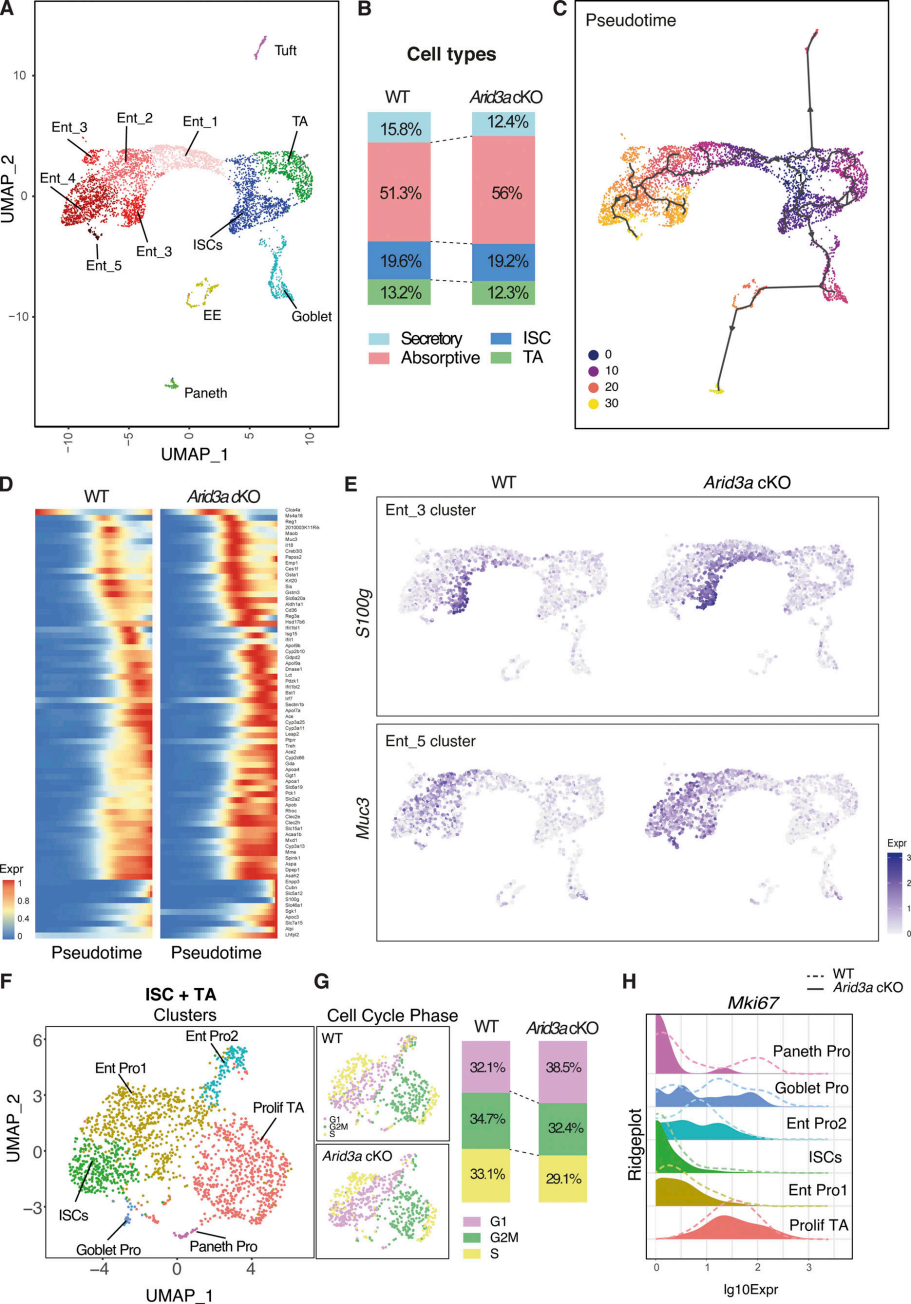
**Single-cell RNA-sequencing reveals changes in enterocyte differentiation trajectory and cell cycle phase in TA cells. (A)** UMAP plot of epithelial cells from WT and *Arid3a* cKO intestine and post-hoc annotation of different cell types. **(B)** Proportion of intestinal cell types in WT and *Arid3a* cKO animals based on scRNA-seq. **(C)** Pseudotime analysis of the scRNA-seq data. ISCs were chosen as the starting point of the trajectory analysis. **(D)** Array of gene expression over pseudotime of the most DEGs that are significant in the enterocyte clusters based on RNA-seq data. **(E)** Feature plot of *S100g* (enterocyte marker cluster 3) and *Muc3* (enterocyte marker cluster 4) expression on UMAP. **(F)** UMAP plot of ISC+TA cells from WT and *Arid3a* cKO intestine and post-hoc annotation of different sub-populations within this compartment. **(G)** Feature plot of cell cycle phase on UMAP of the ISC+TA compartment of WT and *Arid3a* cKO. On the right, proportion in percentages of cells in G1, G2/M, or S phase in the WT and *Arid3a* cKO intestines. **(H)** Ridgeplot showing log10 expression of *Mki67* gene in WT and *Arid3a* cKO for each crypt cell sub-population.

Our bulk transcriptomics analysis in combination with tissue analysis revealed changes in stem-progenitor cell proliferation and cell type composition in the *Arid3a* cKO intestine. To better understand how ARID3A regulates the dynamics of cell proliferation and differentiation, we further performed single-cell RNA-sequencing (scRNA-seq) on the intestinal epithelium from the WT and mutant animals. Clustering of epithelial cells from both WT and *Arid3a* cKO intestine showed 11 major cell clusters including all the known epithelial cell types and curated cell type annotation was performed based on previously published markers of each cell population (Fig. S3 A and Table S2). UMAP analysis further revealed five distinct enterocytes clusters that exhibit similarities with the previously described zonated clusters along the villus axis (Moor et al., 2018) (Fig. 5 A). In accordance with the results observed earlier, we found that loss of *Arid3a* resulted in an increase in enterocyte populations (WT = 51.3%, cKO = 56%) and a reduction of secretory cells (WT = 15.8%, cKO = 12.4%) (Fig. 5 B).

To better understand the changes in differentiation and cell fate dynamics, we performed pseudotime trajectory analysis which revealed three distinct major trajectories: differentiation trajectory toward (1) enterocyte lineage, (2) Goblet, enteroendocrine, and Paneth cells, and (3) Tuft cells (Fig. 5 C). This indicates that Tuft cells are a distinct cell lineage from other secretory cell types. Although the loss of *Arid3a* did not cause major alteration to the overall differentiation dynamics, spatial mapping of the most differentially expressed enterocyte genes (based on the bulk RNA-seq) against pseudotime showed enriched expression of these genes toward earlier pseudo-timeline in the absence of *Arid3a* (Fig. 5 D). This was confirmed by mapping the expression of selected enterocyte markers (*S100g*, *Muc3*, *Ms4a18*, and *Ace2*) in UMAP, which showed increased expression of these genes in the *Arid3a* cKO cells aligned at earlier pseudotemporal trajectory compared with WT (Fig. 5 E and Fig. S3 B), suggesting a role of *Arid3a* in enterocyte differentiation dynamics.

We have previously shown reduced crypt proliferation in *Arid3a* cKO intestine. To further understand the proliferative changes in different crypt-cell populations, ISCs and TA cell clusters were subjected to additional subclustering analysis. This resulted in six cell clusters, including ISCs, proliferative TA cells, Paneth progenitors, Goblet progenitors, and two enterocyte progenitor populations (Fig. 5 F). Cell cycle analysis of these cells showed an overall increase in the G1 phase (WT = 32.1%, cKO = 38.5%) and a corresponding decrease in G2M (WT = 34.7%, cKO = 32.4%) and S phase (WT = 33.1%, cKO = 29.1%) in the mutant crypt cells (Fig. 5 G). Consistent with the EdU quantitation result observed earlier, we noted reduced expression of proliferation markers (*Mki67*, *Pcna*, and *Ccna2*) in different crypt-cell populations of the mutant animals, notably more prominent in the proliferative TA cells (Fig. 5 H and Fig. S3 C).

Together, our data shows that *Arid3a* deletion resulted in reduced proliferation in TA progenitor cells and enhanced enterocyte differentiation. Transcriptomic analysis using both bulk and scRNA-seq indicates that ARID3A modulates intestinal differentiation dynamics in the villus by fine-tuning the expression level of the spatial differentiation markers at the corresponding zones. The results suggest that the increased enterocyte differentiation upon ARID3A loss is caused by an overall increased expression of enterocyte genes as well as a moderate shift of differentiation trajectory.

### Increased binding and transcription of HNF1 and HNF4 in *Arid3a*-depleted intestine

**Figure 6.**
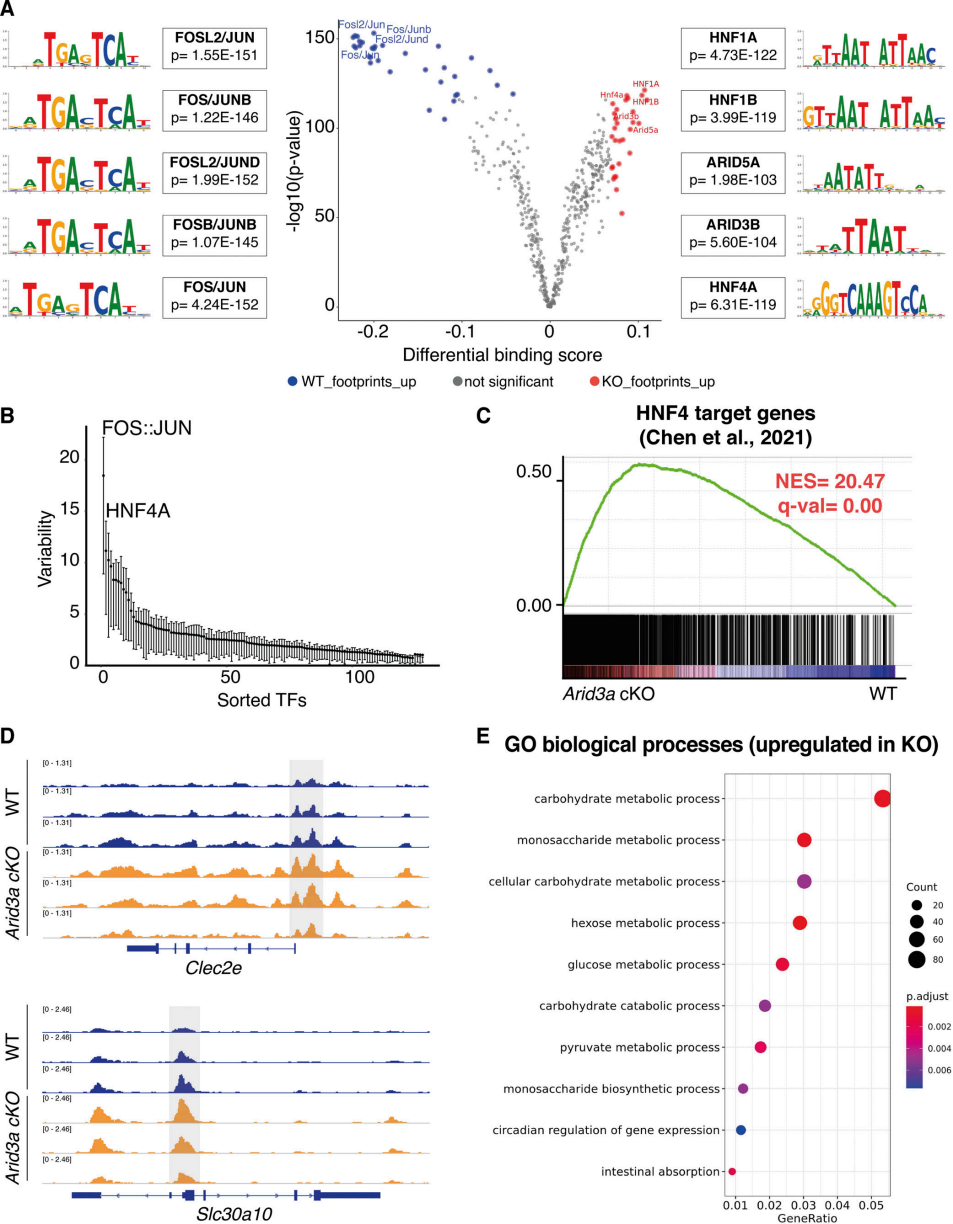
**Deletion of Arid3a allows HNF family of transcription factors to bind to A+T-rich regions. (A)** Analysis of the ATAC-seq using the TOBIAS package (see Materials and methods). Volcano plot shows the differential binding activity against the −log_10_(P value) of all investigated transcription factor motifs. Each dot represents one motif; blue dots represent motif enrichment in WT; red dots represent motif enrichment in *Arid3a* cKO. Representative examples of Top-10 transcription factors enriched in either WT or *Arid3a* cKO animals are shown on the left and right side of the volcano plot, respectively. **(B)** Analysis of the ATAC-seq using the ChromVAR package (see Materials and methods). Plot shows TFs sorted based on their variability score. **(C)** GSEA of previously published gene list of HNF4 target genes. **(D)** Comparison of open ATAC-seq chromatin peaks of Hnf4 targets genes (*Clec2e* and Slc30a10) in WT and *Arid3a* cKO animals (tracks extracted from IGV). **(E)** Top 20 upregulated GO biological processes based on ATAC-seq analysis. GO was performed based on 5,000 peaks with the greatest variability between WT and KO.

To understand how the loss of *Arid3a* causes changes in the cell composition of the intestinal epithelium, we performed ATAC-seq to assess the differences in chromatin accessibility between WT and *Arid3a* cKO intestine. In particular, we performed footprinting analysis of our ATAC-seq using a recently published methodology—Transcription factor Occupancy prediction By Investigation of ATAC-seq Signal (TOBIAS) (Bentsen et al., 2020), which enables genome-wide analysis of transcription factor (TF) dynamics and calculates enriched motif binding using publicly available binding motifs of hundreds of transcription factors. Interestingly, TOBIAS analysis of the ATAC-seq data showed enrichment of transcription factor binding sites in AT-rich genomic regions of the Arid3a cKO intestine (Fig. 6 A). These included members of the ARID family (ARID3B and ARID5A) as well as members of the HNF family (Hnf1 and Hnf4). On the other side, the WT intestine showed enrichment of binding motifs of FOS/JUN dimers (AP-1 pathway) (Fig. 6 A), which has been previously linked with proliferation, apoptosis, and carcinogenesis (Karin et al., 1997). To confirm this result, we utilized ChromVAR to assess TF-associated chromatin accessibility. TFs with a variability score >5 are associated with dynamic chromatin contributing to phenotypic changes, while TFs with a variability score <5 are associated with permissive chromatin (Schep et al., 2017). In accordance with the TOBIAS analysis, FOS/JUN dimers and HNF4A-associated motifs were at the top two highest variability scores (Fig. 6 B), indicating that changes of these TF dynamics contribute to the altered proliferation and differentiation in *Arid3a* cKO intestine.

Interestingly, HNF proteins have been previously associated with the terminal maturation of enterocytes (Babeu et al., 2009; Chen et al., 2019; D’Angelo et al., 2010). It is conceivable that enrichment of HNF activity in the *Arid3a* cKO intestine results in increased spatial differentiation of enterocytes along the villus. Indeed, GSEA of the bulk RNA-seq data showed a strong enrichment of the previously published Hnf4a/g target genes in the *Arid3a* cKO intestine (Fig. 6 C), confirming the increased HNF4 transcription in the absence of *Arid3a*. In accordance with this finding, previously described intestinal epithelial-specific HNF4 targets (*Clec2e* and *Slc30a10*), showed higher chromatin accessibility in the promoter regions of the *Arid3a*-deficient intestine compared with WT (Fig. 6 D). We further performed GO analysis of the 5,000 most variable peaks between WT and *Arid3a* cKO intestine to evaluate the biological processes behind it. Interestingly, we observed significant upregulation of many processes related to enterocyte functions in the cKO intestine including carbohydrate/monosaccharide metabolic processes and intestinal absorption (Fig. 6 E), supporting the notion that HNF-mediated terminal differentiation of enterocytes is enriched in the mutant. Together with the transcriptomic data, we propose that ARID3A functions to maintain cell proliferation and prevent premature HNF-mediated terminal differentiation at the TA progenitor cells.

### Loss of *Arid3a* impairs irradiation-induced regeneration

**Figure S4.**
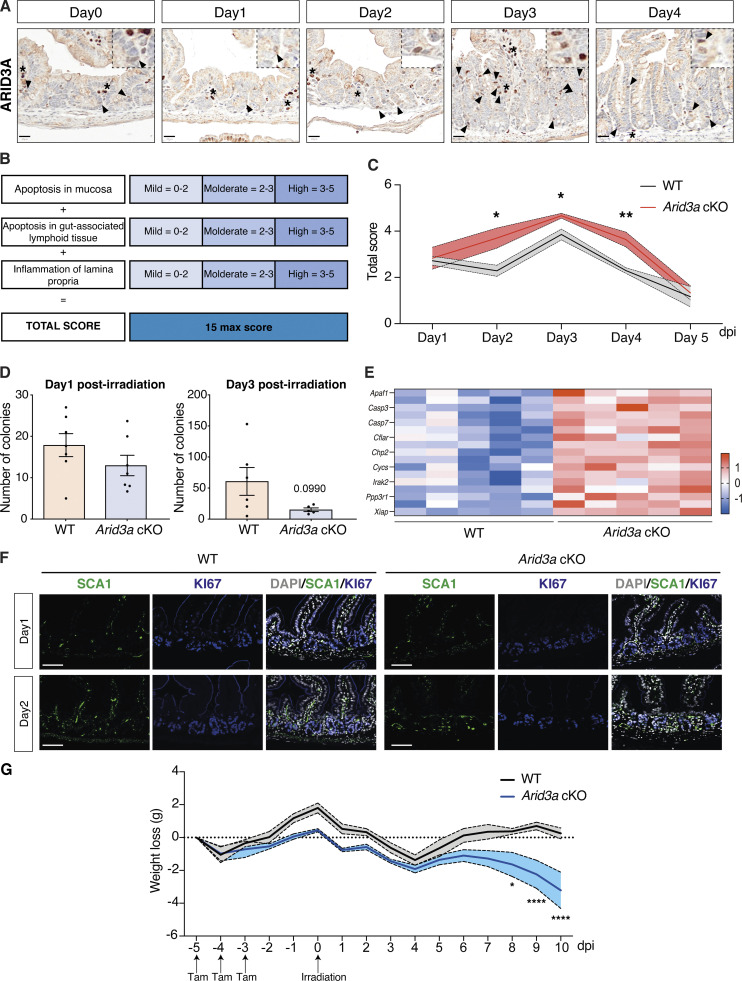
**Loss of Arid3a leads to reduced proliferation and extended tissue damage after irradiation. (A)** ARID3A immunostaining of WT mice during the first 4 days after administration of 12 Gy irradiation. Representative images of *N* = 5 WT animals per time point. Scale bar, 20 μm. Black arrowheads indicate epithelial expression of ARID3A; asterisks indicate stromal expression of ARID3A. **(B)** Description of the scoring system used to assess damage of the small intestine at days. **(C)** Quantification of tissue damage. Data represent mean ± SEM (light color area); *P < 0.05, **P < 0.01, ***P < 0.001, two-sided *t* test. **(D)** Organoid formation assay was performed at days 1 and 3 after irradiation. Data represent mean ± SEM *P < 0.05, **P < 0.01, ***P < 0.001, two-sided *t* test. **(E)** Heatmap of RNA-seq data of representative cell death-associated markers. Z-scores are shown. **(F)** Immunostaining of SCA1 (green) and KI67 (blue) in WT and *Arid3a* cKO animals at days 1 and 2 post-irradiation. Representative images of *N* = 3 animals per genotype. Scale bar, 100 μm. **(G)** Weight changes in WT and Arid3a cKO animals from day 5 (first tamoxifen injection) through day 10 after irradiation. Data represent mean ± SEM; *P < 0.05, **P < 0.01, ***P < 0.001, two-sided *t* test.

Since deletion of *Arid3a* inhibits proliferation of the TA progenitor cells, we asked whether this would affect the regenerative capacity of the intestine upon irradiation. Intestinal epithelium regenerates rapidly within days after irradiation: (1) apoptotic phase (1–2 days post-irradiation, dpi), (2) hyperproliferating/regenerating phase (3–4 dpi), and (3) normalization phase (5 dpi onwards) (Kim et al., 2017). We have shown earlier that *Arid3a* forms an expression gradient from the villus tip to the early progenitor cells at the crypt (Fig. 1). We first asked if ARID3A expression is changed upon irradiation. To address that, we irradiated WT mice and collected the irradiated intestinal tissues at 1, 2, 3, and 4 dpi as well as the non-irradiated controls. Immunohistochemistry analysis showed collapsed crypts and transient loss of *Arid3a*^+^ cells on 1 dpi, followed by crypt elongation and increased numbers of *Arid3a*^+^ cells at the upper crypt during the regenerating phase (3 dpi). Approaching the normalization phase on day 4, the number of ARID3A^+^ cells returned to homeostatic levels (Fig. S4 A). This suggests that *Arid3a* may play a role in intestinal regeneration and restoration of tissue homeostasis after injury.

**Figure 7.**
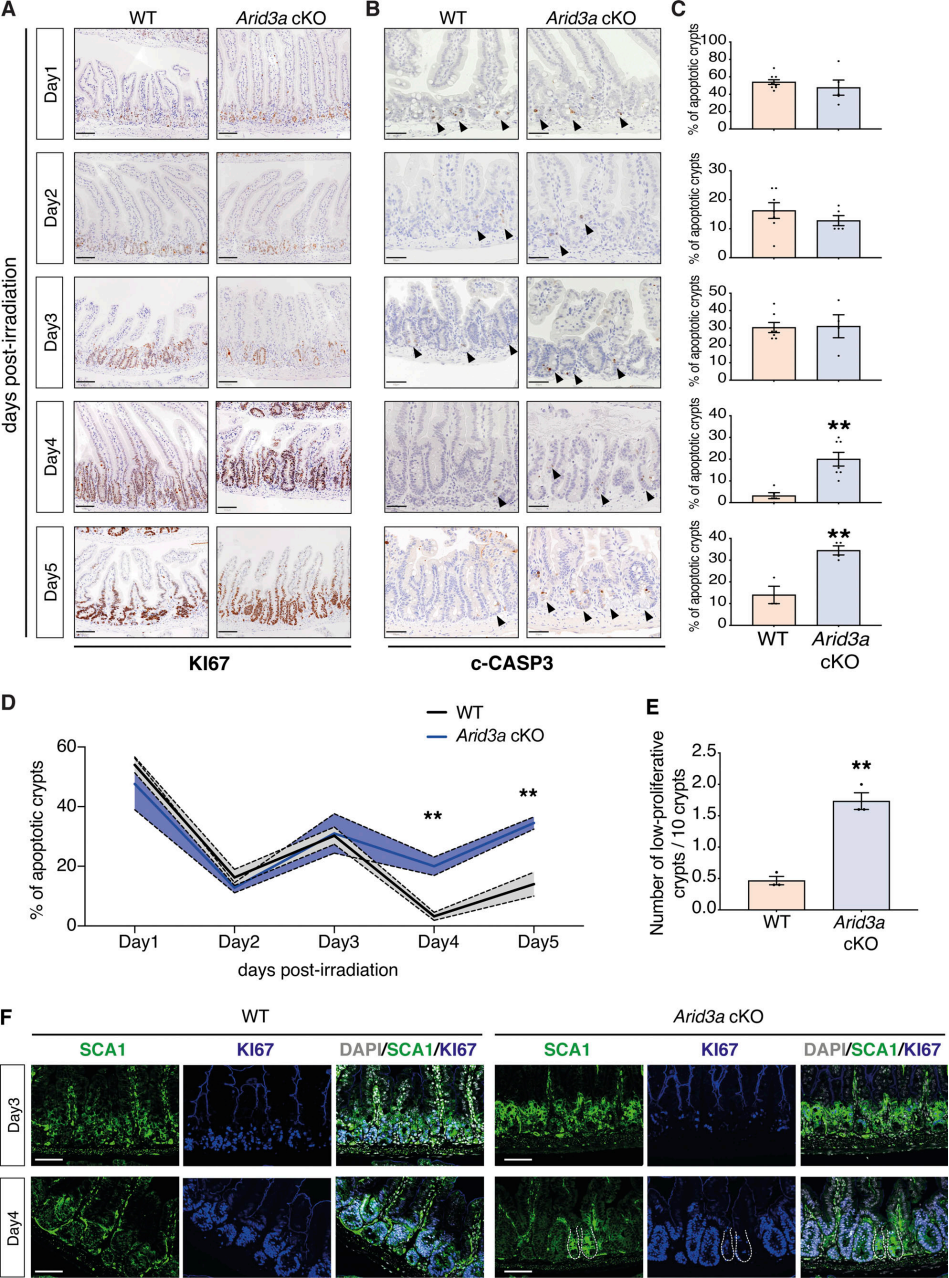
***Arid3a* cKO animals exhibit a delayed irradiation-induced regeneration. (A)** KI67 immunostaining of WT and *Arid3a* cKO mice. Representative images of at least *N* = 4 animals for each genotype per time point. Scale bar, 100 μm. **(B)** c-CASP3 immunostaining of WT and *Arid3a* cKO mice. Representative images of at least *N* = 4 animals for each genotype per time point. Scale bar, 50 μm. Black arrowheads indicate apoptotic cells. **(C)** Quantification of apoptotic crypts for each time point is shown on the right side. Data represent mean ± SEM *P < 0.05, **P < 0.01, ***P < 0.001, two-sided *t* test. **(D)** Summary of quantification of apoptotic crypts in WT and Arid3a cKO animals shown in C. Data represent mean ± SEM (light color area); *P < 0.05, **P < 0.01, ***P < 0.001, two-sided *t* test. **(E)** Quantification of KI67-low crypts in WT and *Arid3a*cKO animals. Quantification of *N* = 3 animals per genotype, 50 crypts per animal. Data represent mean ± SEM *P < 0.05, **P < 0.01, ***P < 0.001, two-sided *t* test. **(F)** Immunostaining of SCA1 (green) and KI67 (blue) in WT an *Arid3a* cKO animals at days 3 and 4 post-irradiation. Representative images of *N* = 3 animals per genotype. Scale bar, 100 μm.

Next, we investigated whether deletion of *Arid3a* perturbs the regenerative response. Expert histological analysis on the irradiated tissues was performed by a local pathologist to assess the damage of the lamina propria, mucosa, and gut-associated lymphoid tissue, and confirmed more extensive tissue damage of cKO animals from day 2 to day 4 when compared with WT (Fig. S4, B and C). We then performed immunostaining of KI67 and cleaved caspase-3 (c-CASP3) to assess tissue proliferation and apoptosis, respectively (Fig. 7, A and B). No major differences were observed between WT and cKO animals on 1 dpi, where extended cell death led to a dramatic reduction of proliferation. On 2 and 3 dpi, the WT intestine started regenerating as evidenced by crypt expansion and increased proliferation, whilst *Arid3a* cKO intestine showed much lower numbers of KI67^+^ cells with minimal crypt expansion. By 5 dpi, the WT intestine had returned to the normalization phase, whereas the majority of *Arid3a* cKO crypts were still in the hyperproliferation phase with elongated crypts (Fig. 7 A). To confirm the reduced proliferative capacity of crypts at the regenerative phase, we isolated crypts from WT and *Arid3a* cKO intestine collected on 1 and 3 dpi and performed organoid formation assay. No differences were observed on day 1 apoptotic phase where organoid formation efficiency was <10% (Fig. S4 D). However, on 3 dpi, most of the WT crypts had restored their capacity to form organoids while the colony formation efficiency remained low for the *Arid3a*-depleted crypts (P value = 0.099) (Fig. S4 D). This is consistent with the earlier observation that the regeneration phase of *Arid3a* cKO intestine was impaired on 3 dpi.

Interestingly, c-CASP3 staining showed no differences in the number of apoptotic cells between WT and mutant at the initial apoptotic and regenerating phase (1–3 dpi) (Fig. 7, B and C), suggesting that perturbation of regeneration was not directly caused by increased apoptosis. However, approaching the normalization phase (4–5 dpi), *Arid3a* cKO intestine showed a significantly higher percentage of apoptotic crypts compared with the control (Fig. 7, B and C). It is interesting to note that the number of apoptotic crypts in both WT and mutant intestines appeared to decrease gradually over time in a wave rather than a linear pattern (Fig. 7 D), suggesting that crypts were unable to regenerate in the first wave after irradiation may collapse again. On the other hand, the apoptotic wave in the *Arid3a*-depleted intestine failed to subside and remained high over time (Fig. 7 D). Of note, increased expression of apoptotic markers was also observed in the cKO intestine during homeostasis, suggesting that ARID3A may also play a role in regulating apoptosis (Fig. S4 E).

The high percentage of apoptotic crypts at 4 and 5 dpi in the mutant intestine suggests impaired crypt regeneration upon loss of ARID3A. Indeed, significantly higher numbers of low-proliferative crypts were observed in the mutant intestine (cKO mean = 1.73 in 10 crypts, WT mean = 0.46 in 10 crypts) during the regenerative phase (4 dpi) as indicated by Ki67 expression (<15 Ki67^+^ cells per crypt) (Fig. 7 E). To characterize the crypt regeneration, we further examined the expression of the fetal marker SCA1 that was induced upon injury (Yui et al., 2018). SCA1 expression was upregulated at 3 and 4 dpi in WT and mutant intestine (Fig. 7 F and Fig. S4 F). However, although crypt proliferation was generally restored at 4 dpi in both WT and mutant intestine, we noted the presence of SCA1^high^ KI67^low^ crypts in the *Arid3a* cKO intestine (Fig. 7 F). This was accompanied by increased weight loss in the *Arid3a* cKO animals over time (Fig. S4 G), indicating that crypt regeneration is impaired in the mutant animals.

Altogether, our findings indicate that Arid3a depletion drives the cellular states of the TA progenitors toward differentiation over proliferation, leading to impaired irradiation-induced intestinal regeneration with sustained cell death and fetal reprogramming activation in the crypts.

## Discussion

ARID3A is a transcription factor with a DNA binding domain that interacts with A+T-rich genomic regions (Herrscher et al., 1995; Patsialou et al., 2005). Functionally, ARID3A has been shown to drive normal development of both myeloid and B cell lineage specification, whereas deletion of the mouse *Arid3a* leads to embryonic lethality due to defective hematopoiesis (Ratliff et al., 2016). Interestingly, *Arid3a* also regulates B-cell response to antigens via posttranslational palmitoylation of cytoplasmic ARID3A, leading to lipid rafts accumulation and B-cell antigen receptor (BCR) signaling (Schmidt et al., 2009). Moreover, ARID3A is enriched in megakaryocytes compared with hematopoietic progenitor cells and has been shown to promote terminal megakaryocytic differentiation (Alejo-Valle et al., 2022). However, the role of ARID3A in intestinal epithelium has not yet been explored. A recent single-cell analysis of the developing gut has identified *Arid3a* as one of the key regulators of intestinal epithelial development through transcription factor regulatory network mapping, whilst the mechanism remains unknown (Fawkner-Corbett et al., 2021).

Here, we report a previously unrecognized role of ARID3A in adult intestinal epithelial differentiation and regeneration. We showed that ARID3A is regulated by WNT and TGF-β signaling to generate an expression gradient from the villus tip to TA cells at the crypt. Loss of *Arid3a* in the intestinal epithelium leads to reduced crypt progenitor cell proliferation and increased enterocyte differentiation, suggesting that ARID3A plays an important role in coordinating the proliferation–differentiation ratio at the intestinal crypt progenitor cells. Expression of ARID3A in the villi may also contribute to the fine-tuning of the spatial differentiation program along the villus by regulating their expression level. Increased binding and transcription of the downstream targets of HNF4 in the mutant intestine suggest that ARID3A may function to maintain TA cell proliferation by inhibiting HNF4-mediated differentiation. Although *Arid3a* deletion promotes enterocyte differentiation and inhibits TA cell proliferation, this does not cause any major phenotypic changes during homeostasis. On the other hand, the reduced proliferating progenitor populations in the mutant intestine impair the irradiation-induced intestinal regenerative response, leading to increased cell death and sustained fetal reprogramming activation in the crypts. We believe that ARID3A functions to fine-tune the proliferation and differentiation dynamics at the crypt progenitors to regulate cell type composition under homeostasis, and these processes are important for injury-induced intestinal regeneration.

It is well understood that TA cells in the intestinal crypts contain progenitors that will continue to proliferate, fueling the intestinal epithelium in the villus, whilst undergoing differentiation in parallel to adopt one of the functional cell types (Beumer and Clevers, 2021). However, the molecular control of proliferation–differentiation switch in TA cells has not been formally characterized. Previous studies of the intestinal epithelium focused mostly on intestinal stem cells and the NOTCH-dependent binary fate decision at the early +4/+5 cell progenitors, while regulation of TA cells is largely overlooked. A recent study showed that modulation of TA cell proliferation changes the balance of absorptive to secretory cell ratio (Sanman et al., 2021), further highlighting the central role of TA cell regulation in intestinal homeostasis. Our current findings imply a new cell-intrinsic regulatory mechanism of TA cells, where ARID3A functions to coordinate the proliferation–differentiation ratio by regulating HNF4 binding and its target gene transcription.

Besides the binary cell fate decision at the early progenitors, it has been recently shown that both absorptive and secretory cells undergo spatial differentiation along the villus axis, resulting in regional and functional heterogeneity of all cell types (Manco et al., 2021; Moor et al., 2018). This implies that intestinal differentiation is a continuous process throughout the crypt–villus axis. More recent studies have shown that the zonation patterning of enterocytes is regulated by a BMP signaling gradient or villus tip telocytes (Bahar Halpern et al., 2020; Beumer et al., 2022), while BMP’s downstream target c-MAF has also been reported to act as a regulator of the intestinal villus zonation program (González-Loyola et al., 2022). The overall increased expression of villus gene signatures across all intestinal epithelial cell types in the mutant animals suggests that ARID3A may have an additional role of spatial differentiation in the villi. Since ARID3A is regulated by TGF-β but not BMP, we propose that TGF-β may also play a role in the spatial gene expression program via ARID3A. This highlights the spatial complexity of the gene regulatory network of intestinal epithelial cell differentiation along crypt–villus axis, with BMP regulating enterocyte zonations and TGF-β regulating the overall spatial differentiation of both lineages. Conceivably, ARID3A facilitates cell fate decision in the early progenitor cells by regulating TA cell proliferation–differentiation ratio, whereas its expression in the villi functions to fine tune the TGF-β-induced spatial differentiation of the committed cells by regulating the expression levels of the zonated genes. It is worth noting that our current model deletes ARID3A in the entire intestinal epithelium, which could not rule out the possibility that loss of ARID3A in differentiated cells may signal back to TA cells. It would be interesting to further explore the potential bidirectional role of ARID3A in both differentiated cells in the villi and TA cells. Indeed, our data indicate that ARID3A promotes Paneth cell differentiation at the early progenitors, which then migrate to the crypt bottom to support ISC maintenance by secreting Wnt ligands. On the other hand, Wnt inhibits ARID3A expression, which in turn limits Paneth cell differentiation and its subsequent Wnt ligand secretion. This would provide a feedback loop to achieve the “just right” level of the Wnt activity at the crypt base by controlling Paneth cell numbers via ARID3A.

Our data further shows the role of ARID3A in intestinal regeneration. It is believed that progenitor cells in the crypt are highly plastic to allow dedifferentiation into regenerative ISCs upon injury. Deletion of *Arid3a* switches the cellular states of the crypt progenitors toward differentiation over proliferation, which results in reduced TA cell numbers and hence plasticity. This will slow down the regeneration process with an extended apoptotic phase and delayed irradiation-induced regeneration, which may resolve over time at a slower and variable pace. This suggests that ARID3A plays an important role in regulating the proliferation-to-differentiation ratio in the progenitor cells for tissue homeostasis and plasticity. Further investigation of the transcription factor network of ARID3A in the intestinal crypt–villus axis would help understand its unique role in fine-tuning the proliferation–differentiation switch.

## Materials and methods

### Animals, drug administration, and treatments

All mouse experiments were performed in accordance with the guidelines of the Animal Care and Use Committee at the Francis Crick Institute. All animals were maintained with appropriate care according to the United Kingdom Animal Scientific Procedures Act 1986 and the ethics guidelines. Arid3a floxed mice were obtained from Joan Yuan (Lund University, Lund, Sweden) and Stephen Malin (Karolinska Institute, Solnavägen, Sweden).

Animal genotyping was performed by PCR amplification of genomic DNA extracted from ear punch biopsies taken from mice aged 3 weeks (see primer list in Table S3). Biopsies were first digested in 200 μl lysis buffer (10 mM Tris pH7.5, 100 mM NaCl, 10 mM EDTA, and 0.5% Sarkosyl) at 55°C overnight. 200 ng of DNA was amplified using MyTaq Red Mix (BIO-25043; Bioline) and 0.4 μM forward and reverse primers in a reaction with final volume of 25 μl. After the initial denaturation step (95°C for 1 min), the thermocycler was configured to 35 cycles of 95°C for 30 s, 56–60°C for 30 s (depending on the primer) and 72°C for 1 min per kilobase of amplification, followed by a final 3 min step of extension at 72°C. PCR products were then visualized and size-verified on a 2% wt/vol agarose/TAE electrophoresis gel with 5 ng/ml ethidium bromide. *Villin*Cre-ERT2 animals were genotyped by the Biological Research Facility of the Francis Crick Institute.

To induce conditional deletion, WT and knockout (cKO) animals were injected intraperitoneally with tamoxifen at 1.5 mg/10 g of mouse weight (from a 20 mg/ml stock solution). Mice were culled by schedule 1 procedure (S1K) at the desired time point.

For EdU chasing experiments, 5-ethynyl-2′-deoxyuridine (EdU) (E10187; Life Technologies) was injected intraperitoneally at 0.3 mg/10 g of mouse weight (from a 10 mg/ml stock solution). Mice were culled by S1K at 2 h after EdU injection.

For irradiation induced–injury experiments mice were exposed to controlled 12 Gray (12 Gy) total body ionizing irradiation to induce damage using a Caesium (γ) irradiator. The dosage rate was 0.779 Gy/min. Mice were culled by S1K at the desired time point.

### Cell culture conditions and maintenance

LS174T cells were maintained in DMEM GlutaMAX (10566-01; Gibco) supplemented with 5% FBS (10270106; Gibco) and 100 U/ml penicillin and 100 μg/ml streptomycin (15140122; Gibco). Cells were incubated in a humidified atmosphere of 5% CO_2_ at 37°C. For WNT pharmacological inhibition, cells were seeded 24 h before treatment with LGK974 inhibitor (S7143; Selleck chemicals).

### Cell line immunofluorescence

For immunofluorescence (IF) experiments, LS174T cells were grown on sterilized glass coverslips in 24-well plates. Coverslips were not coated with poly-*L*-lysine before seeding the cells since LS174T cells are very adherent. Cells were fixed with 4% paraformaldehyde (PFA) for 15 min and permeabilized using 0.5% Triton X-100 in PBS for 15 min. Cells were blocked with 1% bovine serum albumin (BSA) for 1 h at room temperature before overnight incubation with primary antibodies at 4°C (see Table S4 for the full list of antibodies). Cells were washed with PBS and incubated with secondary antibodies conjugated to Alexa-Fluor 488 (A32731; Invitrogen) at room temperature for 1 h in the dark. Cells were washed and stained with DAPI and/or Phalloidin-Atto647 (65906; Sigma-Aldrich) for 30 min in the dark. Coverslips were washed and mounted with ProLong Gold Antifade Mountant (P36934; Thermo Fisher Scientific). Images were acquired as z-stacks using a Leica SPE confocal microscope and processed using Fiji.

### Establishment and maintenance of mouse organoid cultures

Organoids were established from freshly isolated adult small intestines as previously described (Gracz et al., 2012). In brief, 2 cm of jejunal small intestinal tissue was opened longitudinally and the villi was scrapped using a glass cover slip. The remaining tissue was incubated in 15 mM EDTA and 1.5 mM DTT at 4°C for 10 min and moved to 15 mM EDTA solution at 37°C for an extra 10 min. Subsequently, the tissue was shaken vigorously for 30 s to release epithelial cells from the basement membrane and the remaining remnant intestinal tissue was removed. Cells were washed once, filtered through a 70-μm cell strainer, and resuspended in Cultrex BME Type 2 RGF Pathclear (3533-01002; Amsbio). All freshly isolated organoids were maintained in either Intesticult medium (#06005; Stem Cell technologies) or in-house made basal medium containing EGF (PMG8043; Invitrogen), NOGGIN, RSPONDIN, and WNT3A (WENR medium), as previously described (Sato et al., 2009). The Rho kinase inhibitor Y-27632 (Y0503; Sigma-Aldrich) was added to the culture during the first week of crypt isolation and single-cell dissociation. For WNT, Notch, TGF-β, and BMP pathway manipulation, organoids were passaged and allowed to recover for 72 h before treatment. For WNT signaling inhibition, organoids were treated for 48 h with either 5 μM of LGK974 inhibitor or 30 μM of LF3 inhibitor (SML1752; Sigma-Aldrich); for Notch signaling inhibition, organoids were treated with 10 μM of DAPT inhibitor (D5942; Sigma-Aldrich) for the indicated time points; for TGF-β signaling manipulation, organoids were treated with 0.1 ng/ml of recombinant TGF-β1 (11412272001; Sigma-Aldrich) for the indicated timepoints; and for BMP signaling manipulation, organoids were treated with 20 ng/ml of recombinant BMP4 (120-05ET; Peprotech) for the indicated time points.

NOGGIN and RSPONDIN conditioned media were generated by HEK293T cells. WNT3A conditioned medium was generated from L cells. All images were acquired using an EVOS FL Cell Imaging System (Life Technologies) and image brightness was adjusted using Adobe Photoshop (exactly the same parameters were applied to all samples of the same experiment).

### Mouse organoids assay

Organoids were established as described in the previous section. For organoid formation assay, crypts were counted using a brightfield microscope and 200 crypts were seeded in 20 μl of Cultrex BME Type 2 RGF Pathclear in individual wells of a 48-well plate and cultured in WENR medium for 5 days until counted. Three technical replicates were performed per animal.

For RSPONDIN withdrawal assay, organoids were passaged and seeded in three 10-μl droplets per well of a 24-well plate. Organoids were allowed to reestablish in a normal ENR medium (5% RSPONDIN) for 48 h. Subsequently, the organoid medium was replaced and the organoids were cultured in an ENR medium containing either 5% or 1% of RSPONDIN.

To detect disaccharide levels in organoids supernatants, organoids were washed twice with PBS and incubated with a 56 mM solution of sucrose for 1 h. Supernatants were collected and frozen until the assay was performed. To detect glucose content, Amplex Red Glucose/Glucose Oxidase Assay Kit (A22189; Invitrogen) was used. Samples were diluted when necessary and incubated with a reaction buffer containing Amplex Red, horseradish peroxidase, and glucose oxidase. Fluorescence was measured in a Tecan microplate reader with an excitation wavelength of 540 nm and fluorescence emission detection at 590 nm. Glucose concentration was assessed using a glucose standard curve from 0 to 200 µM.

### Crypt–villus fractionation

4 cm of jejunal small intestinal tissue was opened longitudinally and the villi was scrapped using a glass coverslip. Villi and the remaining intestinal tissue were transferred into two separate tubes and washed once. Both parts of the small intestine were incubated in 15 mM EDTA and 1.5 mM DTT at 4°C for 10 min and moved to 15 mM EDTA solution at 37°C for an extra 10 min for the isolation of epithelial cells of villi and crypt fragments. Pelleted cells were resuspended in RLT buffer and stored at −80°C before proceeding to RNA extraction.

### Fluorescent-activated cell sorting (FACS) of GFP-positive cells from *Lgr5-EGFP-ires-CreERT2* mice

Crypts were harvested from the proximal jejunum (∼10 cm) as described in Establishment and maintenance of mouse organoid cultures. Crypts were then dissociated by incubating with Collagenase/Dispase (11097113001; Roche) for 20 min at 37°C, followed by 20 min incubation with TrypLE (12604013; Gibco) for 20 min at 37°C. TrypLE was stopped by adding Advanced DMEM (12491015; Gibco) containing 10% fetal bovine serum (FBS) (10270106; Gibco) and dissociated cells were passed through a 20-μm strainer. Cells were stained with DAPI and resuspended in PBS, 0.5% BSA, and 2 mM EDTA. Cells were separated and recollected in Advanced DMEM plus 10% FBS based on GFP intensity. Cell sorting was performed on a BD FACSAria II System.

### RNA isolation from cell lines, organoids, and tissue

RNA was extracted according to the manufacturer’s instructions (74106; Qiagen RNeasy). Harvested cell lines, organoids, or intestinal crypts were resuspended in RLT buffer (provided with the kit) supplemented with 40 mM dithiothreitol (DTT) to inhibit RNase activity. Before RNA extraction, samples underwent one freeze/thaw cycle to increase the yield of extracted RNA.

### cDNA synthesis and qRT-PCR

500–1,000 ng of RNA were reverse transcribed using the cDNA Reverse Transcription Kit (#4368813; Applied Biosystems) according to the manufacturer’s instructions. RT-qPCR was performed in 384-well plates, in experimental triplicates, in a 12 μl reaction mixture containing 6 μl of 2x PowerUp SYBR Green Master Mix (A25742; Applied Biosystems), 10 μM of each primer, and 25–50 ng of cDNA. The reaction mixture without a cDNA template was used as a negative control for each reaction plate. After 40 cycles of amplification, samples were normalized to housekeeping genes *Ppib* (for mouse samples) or *β-ACTIN* (for human samples), where data was expressed as mean ± SEM.

### Immunohistochemistry

For analysis of the small intestine by immunohistochemistry (IHC), tissues were fixed in 10% formalin and embedded in paraffin. Sections were deparaffinized with xylene and rehydrated in a graded series of ethanol. Antigen-retrieval was performed for 20 min at high temperature in 0.01 M Citrate (pH 6) or Tris-EDTA buffer (10 mM Tris base and 1 mM EDTA, pH 9). Slides were then blocked using the appropriate blocking buffer (10% normal goat serum or 10% normal donkey serum in 1% BSA) and incubated overnight with the appropriate antibody at 4°C (See Table S4 for the full list of antibodies). Finally, slides were incubated with the secondary antibody for 1 h and washed three times with PBS. For colorimetric staining with diaminobenzidine (DAB), slides were incubated with peroxidase substrate, dehydrated, counterstained with hematoxylin solution according to Mayer (51275; Sigma-Aldrich), and mounted. Slides were scanned using an Olympus VS120 slide scanner and images were processed using QuPath (Bankhead et al., 2017).

For immunofluorescence, slides were incubated with Alexa-Fluor 488 or Alexa-Fluor 568 antibody for 1 h, washed three times with PBS, incubated with 4′,6′-diamidino-2-phenylindole (DAPI) for 15 min to visualize nuclear DNA and mounted with ProLong Gold Antifade Mountant. Images were acquired as z-stacks using a Leica SPE or a Leica SP8 confocal microscope and processed using Fiji. For whole-slide imaging, slides were scanned using an Olympus VS120 slide scanner and images were processed using QuPath (Bankhead et al., 2017).

When indicated, sections were stained for H&E, alkaline phosphatase, and AB-PAS staining. Edu was detected according to the manufacturer’s protocol (Click-iT Plus EdU Alexa-Fluor 555 imaging kit C 10638; Thermo Fisher Scientific) to evaluate proliferating cell numbers. Edu+ cells were quantified in at least 10 crypts per mouse.

### In situ hybridisation

Single-molecule in situ hybridization was performed on mouse intestine according to manufacturer’s instructions (ACD; RNAscope 2.5HD Assay RED [REF 322350] or RNAscope 2.5HD [REF 322436]). The probes used were against *Arid3a* (REF 525721), *Arid3b* (REF 525731), *Atoh1* (REF 408791), *Lgr5* (REF 312171), and *Olfm4* (REF 311831). Briefly, guts were fixed in formalin overnight, paraffin-embedded, and cut into 5-μm thick slices. Target retrieval was performed for 15 min, followed by RNAScope Protease Plus incubation for 24 min on the FFPE Sample Preparation and subsequent amplification steps. For brightfield analysis, slides were counterstained using 50% hematoxylin solution according to Mayer, and for immunofluorescence, slides were incubated with DAPI for 10 min for DNA visualization. Images were acquired with an Olympus VS120 slide scanner and images were processed using QuPath (Bankhead et al., 2017).

For combined RNAscope and immunofluorescence of *Arid3a* with MUC2, CHGA, or LYZ, samples were first stained for *Arid3a* using the red channel of duplex RNAscope kit, followed by antibody immunostaining as described above.

### Bulk RNA-seq sample preparation

Crypts or villi were isolated from 10 cm of mouse jejunal small intestinal tissue as described in Crypt–villus fractionation, and RNA was isolated as described in the RNA isolation section. RNA integrity (RIN) was examined using Bioanalyzer 2100 RNA 6000 Nano kit from Agilent and RIN cut-off was set to 7. For crypt samples, libraries were prepared using KAPA mRNA HyperPrep kit (KK8580) according to the manufacturer’s instructions. For most villus samples, the RIN number was <7 and libraries were prepared with KAPA RNA HyperPrep with RiboErase (KK8561) according to the manufacturer’s instructions.

### Bulk RNA-seq data analysis

Fastq files were processed using the nf-core/RNASeq pipeline (https://doi.org/10.5281/zenodo.4323183, Patel et al., 2020) version 3.0 using the corresponding versions of STAR RSEM to quantify the reads against release 95 of Ensembl GRCm38. These raw counts were then imported into R (R Core Team [2020], https://www.R-project.org/) version 4.03/Bioconductor version 3.12 (Huber et al., 2015). We then used DESeq2 (Love et al., 2014) version 1.30.1 to account for the different size factors between the samples and a generalized linear (negative binomial) model with main effects of arid3a (or arid3b) status and time (as a categorial variable) to find genes that were statistically significantly associated with arid3a (or arid3b) status (Wald test) with a false discovery rate of <0.05.

GSEA was performed using the GSEA desktop software (version 4.1.0) using the following parameters: GSEA Preranked > no collapse of gene symbols > classic enrichment statistic > Chip platform “Mouse_Gene_Symbol_Remapping_Human_Orthologs_MSigDB.v7.5.chip.” GSEA custom lists were obtained from the indicated publications. For Metacore analysis, the online software was used (https://portal.genego.com/). Gene lists of upregulated and downregulated genes were created by using FDR < 0.05 and FC > 1.5 cut-offs. One-click analysis included Pathway Maps and GO Processes.

### ATAC-seq sample preparation

Isolated mouse crypts were dissociated to single cells as described in the FACS of GFP-positive cells from *Lgr5-EGFP-ires-CreERT2* mice section, and single cell numbers and viability were assessed using Trypan blue dye and Neubauer chamber. 25,000 cells per sample were transferred to a fresh tube, pelleted, and incubated in RSB buffer (10 mM Tris-Cl pH 7.4, 10 mM NaCl, and 3 mM MgCl_2_) supplemented with 0.1% vol/vol NP-40 (11332473001; Sigma-Aldrich), 0.1% vol/vol Tween-20 (11332465001; Sigma-Aldrich), and 0.01% Digitonin (G9441; Promega) to isolate intact nuclei. Isolated nuclei were subsequently treated with Tn5 transposase (20034197; Illumina) for 30 min at 37°C with agitation for DNA tagmentation. DNA was immediately purified using Qiagen Mini Elute kit (28004; Qiagen). Purified DNA was subsequently used for library preparation using NEBNext High-Fidelity 2X PCR Master Mix (M0541S; NEB) using primers with Nextera dual indexes in a 20 μl final reaction volume. PCR amplification included 5 min incubation at 72°C, followed by 30 s of DNA denaturation at 98°C and 12 cycles of the following: 98°C for 10 s, 63°C for 30 s, and 72°C for 1 min. PCR products were cleaned-up using Ampure XP beads (A63881; Beckman Coulter) according to the manufacturer’s instructions. The quality of the final DNA library was confirmed on the Agilent Tapestation before the samples were submitted for sequencing.

### ATAC-seq data analysis

The nf-core/atacseq pipeline (version 1.2.1) (Ewels et al., 2020) written in the Nextflow domain-specific language (version 19.10.0) (Di Tommaso et al., 2017) was used to perform the primary analysis of the fastq samples in conjunction with Singularity (version 2.6.0) (Kurtzer et al., 2017). The command “nextflow run nf-core/atacseq -profile crick --input/Path_to_desing/design.csv –fasta Mus_musculus.GRCm38.dna_sm.toplevel.fa --gtf Mus_musculus.GRCm38.95.gtf --gene_bed Mus_musculus.GRCm38.95.bed --macs_gsize 2.6e9 --blacklist mm10.blacklist.bed --narrow_peak -r 1.2.1 -resume” was used.

To summarize, the pipeline performs adapter trimming (Trim Galore!—https://www.bioinformatics.babraham.ac.-uk/projects/trim_galore/), reads alignment (BWA), and filtering (SAMtools) (Li et al., 2009), (BEDTools) (Quinlan and Hall, 2010); BamTools (Barnett et al., 2011); pysam—https://github.com/pysam-developers/pysam; picard-tools—http://broadinstitute.github.io/picard, normalized coverage track generation ([BEDTools] [Quinlan and Hall, 2010]; bedGraphToBigWig [Kent et al., 2010]), peak calling (MACS) (Zhang et al., 2008) and annotation relative to gene features (HOMER) (Heinz et al., 2010), consensus peak set creation (BEDTools), differential binding analysis ([featureCounts] [Liao et al., 2014] R Core Team, DESeq2 [Love et al., 2014]), and extensive QC and version reporting (MultiQC [Ewels et al., 2020], FastQC [Daley and Smith, 2013], deepTools [Ramírez et al., 2016], and ataqv [Orchard et al., 2020]). All data were processed relative to the mouse Ensembl GRCm38 release 95. A set of consensus peaks was created by selecting peaks that appear in at least one sample. Counts per peak per sample were then imported on DESeq2 within R environment for differential expression analysis. Pairwise comparisons between genotypes in each condition and between conditions per genotype were carried out, and the differential accessible peaks were selected with an FDR < 0.05.

For footprinting analysis, TOBIAS (v 0.12.10) (Bentsen et al., 2020) was used by running the following pipeline (https://github.com/luslab/briscoe-nf-tobias). The pipeline runs TOBIAS’ ATACorrect, ScoreBigwig, and BINDetect, and generates PlotAggregate metaplots on merged replicate bam files. TOBIAS was run on a set of consensus peaks used for the differential analysis (see above). As described before (Bentsen et al., 2020), all TFs with −log_10_(P value) above the 95% quantile or differential binding scores smaller/larger than the 5% and 95% quantile were colored. Selected TFs are also shown with labels.

For ChromVar analysis, the previously published R package was used (http://www.github.com/GreenleafLab/chromVAR) to analyze sparse chromatin-accessibility data by estimating gain or loss of accessibility within peaks sharing the same motif or annotation while controlling for technical biases. Identified TFs were sorted based on their variability score.

For GO analysis, the 5,000 peaks with the highest variability between WT and *Arid3a* cKO samples were chosen, and the peaks were annotated with the closest gene prior to the analysis.

### scRNA-seq sample preparation

Isolated mouse crypts were dissociated into single cells as described in the FACS of GFP-positive cells from *Lgr5-EGFP-ires-CreERT2* mice section, and single cell numbers and viability were assessed using Trypan blue dye and Neubauer chamber. 3 × 10^6^ cells per sample were stained for CD45-FITC (103108; Biolegend), Epcam-APC (118214; Biolegend), and CD31-PE (102507; Biolegend) at 1/100 dilution during 30 min at RT in the dark. DAPI was added at a 1/1,000 dilution for the last 5 min. After antibody incubation and washing, samples were resuspended in FACS buffer (PBS, 0.5% BSA, 2 mM EDTA). Single-cell stainings and unstained controls were used for gating. Cells were sorted for DAPI-negative to select for live cells, Epcam-positive to acquire epithelial cells, CD45-negative to exclude lymphocytes, and CD31-negative to exclude endothelial cells. After sorting, cells were pelleted and resuspended in 1,000 cells/μl final concentration, and the cell viability was assessed as a quality check parameter. Only samples with viabilities >85% were processed for library preparation, which was done using 10X_3primer_mRNA as per the manufacturer’s instructions. Samples were sequenced using Novaseq platform. A minimum of 5,000 k reads per cell was reached and paired-end sequencing was performed.

### scRNA-seq data analysis

Raw sequencing data were processed using the CellRanger pipeline (version 5.0; 10X Genomics) (Zheng et al., 2017). Count tables were loaded into R (version 4.0.3) and processed using the Seurat R-package for single-cell analysis (version 4.0.5) (Hao et al., 2021). All cells with fewer than 1,000 distinct genes observed per cell and cells with >15% of unique molecular identifiers stemming from mitochondrial genes were removed as low-quality cells prior to the downstream analysis. Integration of individual samples was done using the canonical correlation analysis method from the Seurat package with 3,000 integration features. Principal component analysis was performed on the 2,000 most variable genes in the dataset and the first 30 principal components were selected as input for UMAP dimensionality reduction and clustering. Clustering was performed with the default method of the Seurat R-package, with the resolution parameter set to 0.3. Cluster marker genes were inspected, and the corresponding cell types were assigned manually. Single-cell differential gene expression analyses were performed using the R-package glmGamPoi (version 1.2.0) (Ahlmann-Eltze and Huber, 2021). Trajectory analyses were conducted using the monocle3 R-package (version 0.2.2) (Cao et al., 2019) with the ISC cluster 0 set as a starting point. A sub-clustering of cells in the ISC and TA clusters was performed. For the sub-clustering analysis, the cluster resolution parameter was set to 0.3 and clusters were assigned manually. Cell cycle phases were estimated using the CellCycleScoring function of the Seurat R-package.

### Online supplemental material

Fig. S1 shows additional characterization of Arid3a cKO intestine. Fig. S2 shows an additional analysis of the bulk RNAseq data of the mutant intestine. Fig. S3 shows additional analysis of the scRNAseq data of the mutant intestine. Fig. S4 shows additional analysis of Arid3a cKO intestine upon irradiation. Table S1 shows differential gene expression analysis of Arid3a cKO crypts versus WT. Table S2 shows cluster-specific gene signatures based on scRNA-seq analysis. Table S3 lists the of primers used in this study. Table S4 is the list of antibodies used in this study.

## Supplementary Material

Table S1 shows differential gene expression analysis of Arid3a cKO crypts versus WT.

Table S2 shows cluster specific gene signatures based on scRNA-seq analysis.

Table S3 is a list of primers used in this study.

Table S4 lists antibodies used in this study.

## Data Availability

The accession numbers for the raw and processed data used for this study were deposited on GEO and is publicly available: Bulk RNA-seq GEO: GSE242983, scRNA-seq GEO: GSE243155, and ATAC-seq GEO: GSE212560.
